# vGPCR-Mediated ANO1 Modulation of Apoptosis Regulates Lytic KSHV Infection

**DOI:** 10.3390/v18090954

**Published:** 2026-08-31

**Authors:** Anisha Reddy Konakalla, Osvaldo K. Moreno, Savannah E. Price, Erica L. Sanchez

**Affiliations:** 1 Department of Biological Sciences, The University of Texas at Dallas, Richardson, TX 75080, USA; anishareddy.konakalla@utdallas.edu (A.R.K.); savannah.price@utdallas.edu (S.E.P.); 2 Department of Molecular and Human Genetics, Baylor College of Medicine, Houston, TX 77030, USA; osvaldo.moreno@bcm.edu

**Keywords:** Kaposi’s Sarcoma Herpesvirus (KSHV), Kaposi’s sarcoma (KS), ANO1, TMEM16A, vGPCR, apoptosis, transcriptomics, lytic replication

## Abstract

Kaposi’s sarcoma-associated herpesvirus (KSHV) is an oncogenic gammaherpesvirus that causes Kaposi’s sarcoma (KS), an endothelial cell-derived malignancy that primarily affects immunocompromised individuals. During lytic reactivation, KSHV expresses viral proteins that promote viral replication and modulate host signaling pathways. Here, we identified the calcium-activated chloride channel ANO1 (TMEM16A) as a previously unrecognized host factor induced by the KSHV lytic protein vGPCR. RNA sequencing, RT-qPCR, and immunofluorescence analyses demonstrated robust ANO1 upregulation in vGPCR-expressing endothelial cells and during KSHV lytic reactivation. siRNA-mediated depletion of vGPCR significantly reduced ANO1 expression, demonstrating that vGPCR contributes to ANO1 induction during infection. Functionally, ANO1 knockdown sensitized vGPCR-expressing endothelial cells to caspase-dependent apoptosis under serum-starved conditions, which was rescued by the pan-caspase inhibitor Z-VAD-FMK. In reactivated iSLK.BAC16 cells, genetic or pharmacological inhibition of ANO1 increased late apoptosis, enhanced KSHV lytic gene expression, and promoted infectious virion production. Together, these findings identify ANO1 as a vGPCR-regulated host factor that promotes cell survival and modulates KSHV lytic replication, highlighting ANO1 signaling as a potential therapeutic target in KSHV-associated disease.

## 1. Introduction

Kaposi’s Sarcoma (KS), caused by Kaposi’s Sarcoma Herpesvirus (KSHV), is an endothelial cell-based tumor and the most common type of cancer in HIV-infected, untreated individuals [1,2]. KSHV is an oncogenic enveloped double-stranded DNA virus and belongs to the *Gammaherpesvirinae* subfamily. KSHV is also associated with lymphoproliferative disorders such as primary effusion lymphoma (PEL), Multicentric Castleman’s Disease (MCD), and KSHV inflammatory cytokine syndrome (KICS) [3]. KSHV infection of endothelial cells results in morphological, metabolic, lifespan, and gene expression changes to facilitate virion production [2,4,5,6].

KSHV pathogenesis exhibits two different modes of infection, latent infection and lytic reactivation. Most KS cells are in the latent phase by default, with only 1–5% lytically replicating [7,8]. The latent phase of KSHV is marked by limited viral gene expression, no virion production, and long-term host cell survival. However, during the lytic phase, the viral episome undergoes chromatin remodeling that permits the sequential expression of immediate-early, early, and late lytic genes, leading to productive replication and production of infectious virions, which can ultimately result in host cell lysis [2]. Lytic viral proteins can drive oncogenesis, induce cellular proliferation, and modulate the host immune system [2]. The KSHV lytic cycle is initiated by expression of the immediate-early gene and trans-regulator Replication and Transcriptional Activator (RTA, ORF50) [9].

Currently, the focus of KSHV research lies predominantly in the latent phase of infection, as latently infected cells comprise the majority of KS tumors and latency allows for long-term viral persistence and immune evasion [10]. However, the lytic replication stage remains underexplored, despite its critical role in viral dissemination, inflammation, and tumor progression. Many lytic genes are expressed during reactivation and act as potent modulators of host signaling pathways that promote various cellular processes of infected cells [11,12,13]. One such lytic gene is the viral G protein-coupled receptor (vGPCR), which is constitutively active during early reactivation and is known to be further activated by several cellular cytokines. vGPCR is a seven-transmembrane signaling protein, homologous to the cellular cytokine receptor CXCR2 [14,15,16,17,18]. Recent omics approaches have substantially expanded our understanding of gammaherpesvirus-host interactions by identifying changes in host and viral transcriptional, proteomic, and metabolic programs during latent and lytic infection [19]. Although extensive work has characterized oncogenic signaling pathways of vGPCR, far less is known about how vGPCR reshapes global host transcriptional programs in endothelial cells or how host factors induced by vGPCR contribute to cell survival and KSHV lytic replication. To address this gap, we combined endothelial vGPCR overexpression with a KSHV-infected iSLK.BAC16 model to identify vGPCR-dependent host genes and determine their functional roles during viral reactivation. Understanding the mechanisms and impact of lytic gene expression can uncover new therapeutic vulnerabilities that are missed when focusing solely on latency.

Transcriptomic analysis revealed several previously unrecognized vGPCR targets that were upregulated, including Anoctamin 1 (ANO1), a transmembrane calcium chloride channel implicated in apoptosis inhibition, which also has a potential role in nucleotide regulation [20,21,22]. We investigated vGPCR-dependent upregulation of ANO1 to better understand the role this cellular protein plays in apoptosis. ANO1 has been shown to induce an anti-apoptotic effect via activation of the PI3K/Akt pathway in cancer models [22]. Interestingly, vGPCR has also been reported to induce an anti-apoptotic effect by activating the PI3K/Akt pathway when overexpressed [23]. However, whether ANO1 is regulated by vGPCR during KSHV infection has not been investigated. Our study addresses this gap by demonstrating that vGPCR contributes to ANO1 upregulation during KSHV infection and by evaluating how ANO1 contributes to apoptosis, lytic gene expression, and infectious virion production.

Building on these studies, we investigated the role of ANO1 in KSHV lytic replication and infectious virion production. Using siRNA-mediated knockdown and pharmacological inhibition with DES and Ani9, we found that loss of ANO1 increased apoptotic cell death and enhanced KSHV lytic gene expression and infectious virion production from iSLK.BAC16 cells. These findings identify ANO1 as a previously unrecognized host factor that modulates apoptosis, KSHV lytic gene expression, and infectious virion production during lytic reactivation. Together, these findings identify ANO1 as a vGPCR-regulated host factor that promotes cell survival and modulates KSHV lytic infection, highlighting host ion channel signaling as a potential therapeutic target in KSHV-associated pathogenesis.

## 2. Materials and Methods

### 2.1. Plasmid Construction

To express SSF-tagged vGPCR in HMEC-1, we generated the plasmid pcDNA3.1(+)-SSF-vGPCR (6453 bp). The pcDNA3.1(+) backbone contains the CMV enhancer and CMV promoter for high-level expression, followed by a T7 promoter and a multiple cloning site (MCS). An N-terminal SSF tag (signal sequence + FLAG epitope) was inserted into the MCS region. Immediately downstream, the full-length vGPCR/ORF74 coding sequence was cloned in frame with the SSF tag, producing an N-terminally tagged SSF-vGPCR fusion protein. The insert region contains two components, the SSF tag (78 bp) and the vGPCR ORF (1029 bp), together making the SSF-vGPCR coding region 1107 bp, with additional flanking homology sequences included during synthesis to enable cloning. The plasmid retains all regulatory elements of pcDNA3.1(+), including the bovine growth hormone polyadenylation signal (bGH pA), SV40 promoter and origin, and AmpR and NeoR/KanR cassettes for bacterial and mammalian selection. Restriction sites such as ClaI, XhoI, BmtI, EcoRI, and XcmI flank the SSF-vGPCR region and were used for cloning and diagnostic digestion.

pcDNA3.1(+) (5428 bp) served as the empty vector control, containing the CMV promoter and standard expression elements without any inserted gene.

### 2.2. Cell Culture

HMEC-1 were maintained in MCDB 131 media (Corning, Corning, NY, USA) 15-100-CV) supplemented with 10% fetal bovine serum (FBS) (Corning, 35-011-CV), 1 μg/mL hydrocortisone (Stem Cell Tech, Vancouver, BC, Canada, 74142), 10 ng/mL epidermal growth factor (Biovision, Milpitas, CA, USA, 4022-500), and 10 mM L-glutamine (Gibco, Grand Island, NY, USA, 25030081) at 37 °C and 5% CO_2_.

iSLK and iSLK.BAC16 cells were maintained at 37 °C and 5% CO2 in Dulbecco’s Modified Eagle Medium (DMEM) media (Gibco, 1195-065) supplemented with 10% FBS (Corning, 30-249-CR), 2 mM L-Glutamine (Gibco, 25030081). iSLK.BAC16 cells were selected with 1 μg/mL puromycin (Mirus, Marietta, GA, USA, 22103916), 250 μg/mL G-418 (Fisher Bioreagents, Waltham, MA, USA, BP673-5), and 50 μg/mL hygromycin (Corning, 30-240-CR) [7,24] to achieve stable latent infection. iSLK and iSLK.BAC16 cells were treated with 1 mM sodium butyrate (NaB) (Sigma Aldrich, St. Louis, MO, USA, #303410) and 1 μg/mL doxycycline hyclate (Dox) (Sigma Aldrich, #D5207) for 48 h to induce lytic replication. For iSLK cells, iSLK media were supplemented with 1 μg/mL puromycin and 250 μg/mL G418. Experiments using iSLK.BAC16 and iSLK cells were performed using Dulbecco’s modified Eagle medium (DMEM) supplemented with 10% FBS and 2 mM L-glutamine.

### 2.3. Transient Transfection

Cells were seeded and grown overnight. HMEC-1 cells were transiently transfected with pcDNA3.1(+)-SSF-vGPCR or empty vector control using the TransIT-X2 Dynamic Delivery System (Mirus, MIR 6000) according to the manufacturer’s protocol. Cells were harvested 48 h post-transfection for downstream analyses.

### 2.4. RNA Extraction and RT-qPCR

HMEC-1 cells were seeded for bulk RNA sample preparation and other analyses. Transfections were performed at ~70% confluency as described previously. Cells were washed with PBS once, and RNA was isolated using the PureLink RNA Extraction kit (Invitrogen, Waltham, MA, USA, 12183025) according to the manufacturer’s protocol. RNA was eluted in RNase-free water, and the concentration of each sample was determined via UV-Vis spectrophotometry. Two-step quantitative real-time reverse transcription-PCR (Bio-Rad, Hercules, CA, USA) was used to measure expression levels of vGPCR and other known targets of vGPCR before sending the samples for sequencing. Relative levels of each transcript were normalized using the delta threshold cycle method to the abundance of GAPDH mRNA.

iSLK and iSLK.BAC16 cells were seeded for bulk RNA sequencing and other analyses. The next day, cells (~70% confluency) were treated with doxycycline (Dox) (1 μg/mL) and sodium butyrate (NaB) (1 μg/mL) for 48 h to induce lytic reactivation and were compared to the untreated cells. After 48 h of Dox and NaB induction, cells were washed with PBS once, and RNA was extracted using the PureLinkTM RNA Mini Kit (Invitrogen, 12183025) according to the manufacturer’s protocol. cDNA was generated from RNA using the iScript cDNA Synthesis Kit (Bio-Rad, 1708841). Briefly, 1 μg of RNA was used per reaction primed. The reverse transcription reaction was performed according to the manufacturer’s protocol. qRT-PCR was performed using a QuantStudio 3 Real-Time PCR Detection System instrument (Applied Biosystems, Waltham, MA, USA). cDNA generated from extracted RNA was used as the input for the qRT-PCR, with amplification by the iTaq Universal SYBR Green Supermix (Bio-Rad, 1725124). Two-step quantitative real-time reverse transcription-PCR (Bio-Rad) was used to measure expression levels of vGPCR and other lytic genes. Relative transcript levels were normalized to tubulin mRNA using the 2^−ΔΔCt^ method.

For graphical presentation, fold-change values were normalized to the corresponding reactivated (Dox/NaB-treated) control group within each experiment unless otherwise indicated. Statistical analyses were performed on the normalized biological replicate values using GraphPad Prism 10.5.0. All samples were tested in triplicate or more. All primers were synthesized by Integrated DNA Technologies, Inc. (San Diego, CA, USA), and their sequences are described in Table 1.

### 2.5. RNA Sequencing

HMEC-1 cells were seeded in 10 cm dishes for bulk RNA sample preparation. Transfections were performed at ~70% confluency, and RNA was extracted after 48 h of transfection as described previously. RNA was sent to NovoGene (Sacramento, CA, USA) for sequencing. Messenger RNA was purified from total RNA using poly-T oligo-attached magnetic beads. After fragmentation, the first strand of cDNA was synthesized using random hexamer primers, followed by the second strand of cDNA synthesis using either dUTP for a directional library or dTTP for a non-directional library. For the non-directional library, it was ready after end repair, A-tailing, adapter ligation, size selection, amplification, and purification. For the directional library, it was ready after end repair, A-tailing, adapter ligation, size selection, USER enzyme digestion, amplification, and purification. The library was checked with Qubit and real-time PCR for quantification and a bioanalyzer for size distribution detection. Quantified libraries were pooled and sequenced on Illumina platforms according to effective library concentration and data amount. The clustering of the index-coded samples was performed according to the manufacturer’s instructions. After cluster generation, the library preparations were sequenced on an Illumina platform, and 150 bp paired-end reads were generated.

iSLK and iSLK.BAC16 cells were seeded in 6-well plates, grown overnight, and induced using Dox and NaB, and RNA was extracted as described previously. RNA samples were purified using DNase 1 Amplification Grade (Invitrogen, 18068015) before sending the samples for sequencing at the UT Dallas Genomic Core. Total RNAs were purified and subjected to Illumina stranded mRNA library preparation according to the manufacturer’s instructions (Illumina, San Diego, CA, USA). The libraries were quantified by Qubit (Invitrogen), and the average size of the libraries was determined using the High Sensitivity NGS fragment analysis kit on the Fragment Analyzer (Agilent, Santa Clara, CA, USA). The normalized libraries were then sequenced on an Illumina NextSeq2000 sequencing platform with 100 bp paired-end reads.

### 2.6. RNA-Seq Data Analysis

#### 2.6.1. Quality Control

Raw data (raw reads) of the FASTQ format were first processed through in-house Perl scripts. In this step, clean data (clean reads) were obtained by removing reads containing adapters, reads containing poly-N, and low-quality reads from the raw data. At the same time, Q20, Q30, and GC content of the clean data were calculated. All the downstream analyses were based on clean data with high quality.

#### 2.6.2. Reads Mapping to the Reference Genome

The human reference genome, GRCh38, and its corresponding annotations were obtained from NCBI Datasets and indexed using the software Spliced Transcripts Alignment to a Reference (STAR) v2.7.11b. Paired-end reads were converted from the BCL file format to the fastq file format using Illumina’s bcl2fastq v2.20 and filtered based on quality before being aligned to the indexed genome using STAR v2.7.11b to produce Bam files.

#### 2.6.3. Quantification of Gene Expression Level

The ‘featureCounts’ function from the Rsubread v2.20 R package was used to count the number of reads mapped to each gene for each Bam file. These counts were normalized to gene length and sequencing depth, calculating the expected number of fragments per kilobase sequenced per million base pairs sequenced (FKPM), using the ‘fkpm’ function from the DESeq2 v1.48.0 R package.

#### 2.6.4. Differential Expression Analysis

Differential expression analysis of two conditions/groups (two independent biological replicates per condition for the HMECs and five independent biological replicates per condition for the iSLKs) was performed using the DESeq2 R package (1.48.0). DESeq2 determines the statistical significance of differential expression through fitting the transcript counts of each gene to a general linear model (GLM) using negative binomial regression. The resulting *p*-values were adjusted using Benjamini and Hochberg’s approach for controlling the false discovery rate. For the HMEC-1 dataset, genes with an adjusted *p*-value < 0.05 and Log2FoldChange > |1| were assigned as differentially expressed. For the iSLK dataset, genes with an adjusted *p*-value < 0.05 and Log2FoldChange > |2| were assigned as differentially expressed.

#### 2.6.5. Visualization and Gene Ontology Analysis

To generate the volcano plots, use the ‘geom_point’ function from the ggplot2 v3.5.2 R package. Heatmaps were generated using the pheatmap v1.0.12 R package. Gene set enrichment analysis for gene ontology terms was performed using the ‘gseGO’ function from the ClusterProfiler v4.16.0 R package, using genes ranked by DESeq2-calculated Wald statistic as input. Results were plotted using ggplot2 v3.5.2’s ‘geom_col’ function.

### 2.7. siRNA Transfections and Serum Starvation

For siRNA transfections, cells were seeded in a 6-well plate and transfected with either pcDNA3.1(+)_SSF-vGPCR or an empty vector using TransIT-X2 (Mirus, MIR 6000). At 24 h post-transfection, cells were transfected with siANO1 (Qiagen, Venlo, Limburg, The Netherlands) or non-targeting scrambled control All-Star Negative siRNA (siASN) (Qiagen, 1027295) at a final concentration of 25 pmol. 36 h post-siRNA transfections, cells were cultured in either complete growth medium or serum-deprived medium supplemented with L-glutamine for 12 h prior to downstream analyses.

iSLK and iSLK.BAC16s cells were transfected with siANO1 (Qiagen) or ASN control siRNA (Qiagen, 1027295) at a final concentration of 25 pmol siRNA, using Lipofectamine RNAiMAX (Invitrogen, 13778150) according to the manufacturer’s protocol. 24 h post-transfection, lytic reactivation was induced for 48 h with doxycycline (1 μg/mL) and sodium butyrate (1 mM), where indicated.

The knockdown of ANO1 was accomplished using FlexiTube GeneSolution siRNA (Qiagen, 1027416) according to the manufacturer’s protocol, with 4 types of siRNA for HMEC-1 cells, and only the TMEM16A_6 ANO1 knockdown efficiency for BAC16 cells was confirmed using RT-qPCR analysis before moving forward with other assays.

For vGPCR knockdown, iSLK.BAC16 cells were transfected with a custom siRNA targeting ORF74 (vGPCR) (sense: 5′-GUUAUGAGAACGCGGGAAAAUU-3′), which was designed and synthesized by Horizon Discovery (Waterbeach, Cambridge, UK) at a final concentration of 50 nM using Lipofectamine RNAiMAX according to the manufacturer’s instructions. A non-targeting negative control siRNA (D2-001810-01-20, Horizon Discovery) was used as the control. Twenty-four hours after transfection, cells were induced into the lytic cycle with doxycycline (1 μg/mL) and sodium butyrate (1 mM) for 48 h before RNA extraction and subsequent analyses. Knockdown efficiency was confirmed by RT-qPCR.

### 2.8. Trypan Blue Cell Viability Assay

Cells were seeded and treated as described in the above section. Culture media were collected, and adherent cells were harvested by trypsinization. Cell suspensions were combined and mixed 1:1 with 0.4% Trypan Blue solution (Life Technologies, Waltham, MA, USA, T10282). Viable and dead cells were counted manually using a hemocytometer under a VWR inverted microscope. Cell death percentage was calculated using the following formula:

Cell death percentage = [(Total # dead cells)/(Total # live cells + Total # dead cells)] × 100

### 2.9. Apoptosis Rescue Experiment

To determine whether cell death was due to apoptosis, a rescue experiment was performed using the Z-Vad pan-caspase inhibitor (Z-VAD-FMK) (Promega, Madison, WI, USA, G7232). Cells were seeded and treated as described above and incubated with the pan-caspase inhibitor Z-VAD-FMK (25 µM) or an equivalent volume of DMSO control during the 12 h serum starvation period. Cell viability was subsequently assessed by Trypan Blue exclusion as described above.

### 2.10. Active Caspase-3 Flow Cytometry Assay

Cleaved caspase-3 activation was assessed using the Caspase-3 (Active) Red Staining Kit (ab65617, Abcam, Cambridge, Cambridgeshire, UK) according to the manufacturer’s instructions. iSLK.BAC16 cells were transfected with either siANO1 or ASN control siRNA and were either left non-reactivated or induced for KSHV lytic reactivation with doxycycline and sodium butyrate (Dox/NaB). As a positive control for apoptosis, cells were treated with 1 μM staurosporine for 4 h. Activated caspase-3 was detected using the cell-permeable Red-DEVD-FMK probe, which irreversibly binds activated caspase-3 in apoptotic cells. Following staining, cells were washed and resuspended in 300 µL of Wash Buffer IV and kept on ice until analysis. Samples were analyzed by flow cytometry using the PE channel, and caspase-3 activation was quantified as the percentage of Red-DEVD-FMK-positive cells. A minimum of 10,000 events were collected per sample, and flow cytometry data were analyzed using FlowJo software (v11.0.2).

### 2.11. Flow Cytometry

HMEC-1 and iSLK.BAC16 cells were seeded and treated as described above. Media were collected, cells were washed once with PBS, and trypsinized and pelleted by centrifugation at 300× *g* for 5 min. The eBioscience™ Annexin V Apoptosis Detection Kit (Invitrogen 88-8007-74, 88-8005-74) was used to detect early and late apoptotic cells. The supernatant was discarded, and the cell pellet was washed once with PBS and once with 1× binding buffer provided in the kit. Cells were then resuspended in a 100 μL 1× binding buffer. 5 μL of Annexin V-FITC/APC was added to the resuspended cells and incubated for 10–15 min at RT. Cells were then washed in 1× binding buffer and then resuspended in 200 μL 1× binding buffer. 5 μL of Propidium Iodide (PI) was added to the resuspended cells. For the flow cytometry analysis, after staining, the samples were analyzed within 30 min to 1 h using a flow cytometer equipped with filters suitable for detecting FITC (excitation/emission 490/525 nm), APC (excitation/emission 650/660 nm), and PE-Texas Red (excitation/emission 535/617 nm) fluorescent dyes. A BD Fortessa SORP cytometer (BD FACSDiva Software v9.0), available at the UT Dallas Biological Sciences core, was used for FACS analysis.

During data collection, a minimum of 10,000 events per sample were recorded. Flow cytometry data were analyzed using FlowJo v11.0.2 and BD FACSDiva software (BD FACSDiva Software v9.0). Cells were initially gated using forward scatter (FSC) and side scatter (SSC) to exclude debris, followed by FSC-A versus FSC-H gating to exclude doublets. Quadrant gates were established individually for each independent experiment based on the fluorescence distribution of that experiment and then applied uniformly to all samples within that experiment. Annexin V^+^/PI^−^ cells (Q1) were classified as early apoptotic, Annexin V^+^/PI^+^ cells (Q2) as early-late apoptotic, Annexin V^−^/PI^+^ cells (Q3) as late apoptotic, and Annexin V^−^/PI^−^ cells (Q4) as viable. For HMEC-1 experiments, total apoptotic cells were calculated as the combined percentage of Annexin V- and/or PI-positive cells (Q1 + Q2 + Q3) to minimize potential overinterpretation of individual apoptotic subpopulations due to FITC spectral spillover into the PI channel. For iSLK.BAC16 experiments, early- and late-apoptotic populations were analyzed separately as indicated in the corresponding figures. Because GFP is constitutively expressed in all iSLK.BAC16 cells, any spectral contribution to the PI channel would be expected to be equivalent across all treatment groups within each experiment and is therefore unlikely to affect comparisons between experimental conditions. Data were exported as raw percentages of each cell population and analyzed using GraphPad Prism 10.5.0 software. Statistical analyses were performed using the raw biological replicate values. All experiments were performed in at least three independent biological replicates, with statistical significance determined using one-way ANOVA.

### 2.12. Immunofluorescence Assay (IFA)

HMEC-1 cells were seeded and transfected with either empty vector (EV) or SSF-vGPCR plasmids as described above. For ANO1 knockdown experiments, cells were transfected with either siANO1 or siASN control. 48 h post-transfection, cells were washed with PBS and fixed with 4% paraformaldehyde (PFA) for 10 min at room temperature, followed by three PBS washes. For iSLK.BAC16 cells, the same immunofluorescence staining protocol was used, except that only ANO1 was detected using the rabbit monoclonal anti-TMEM16A antibody, followed by Alexa Fluor 647 goat anti-rabbit IgG secondary antibody. Cells were reactivated with doxycycline and sodium butyrate for 48 h prior to fixation. Cells were permeabilized with 0.1% Triton X-100 in PBS for 10 min at room temperature and blocked with 1% BSA in PBS for 1 h at room temperature. Cells were incubated with primary antibodies diluted in a blocking buffer for 45 min at room temperature, followed by three PBS washes. Primary antibodies used were the mouse anti-FLAG M1 antibody (Millipore Sigma, Burlington, MA, USA, F3040, 1:750) and the rabbit monoclonal antibody to TMEM16A (Abcam ab64085, 1:100). Cells were then incubated with the appropriate Alexa Fluor-conjugated secondary antibodies, AF568 Goat anti-mouse IgG2b (Invitrogen, A-21144, 1:750) and AF647 Goat anti-Rabbit IgG (Invitrogen, A-21245, 1:300), for 45 min at room temperature protected from light. Following three PBS washes, nuclei were stained with DAPI for 5 min and washed with PBS. Images were acquired using an Evident Scientific FV4000 Confocal Laser Scanning Microscope (cellSens FV v3.2.1.85) equipped with a 60× objective and 1× zoom. DAPI was excited at 405 nm and collected between 430 and 470 nm. Alexa Fluor 568 was excited at 561 nm and collected between 580 and 630 nm. Alexa Fluor 647 was excited at 640 nm and collected between 652 and 713 nm. Identical acquisition settings were maintained across all samples within each experiment. Raw 16-bit images were processed using CellSens software (v4.2.1). Brightness and contrast adjustments were applied uniformly across all samples within each channel. FLAG and ANO1 protein expression was quantified using Fiji/ImageJ (v1.54f) by applying an identical fluorescence threshold across all images and measuring the mean fluorescence intensity of the thresholded FLAG and ANO1-positive signal within the entire field of view. Quantification was performed on images from four independent biological replicates, and mean fluorescence intensity values were used for statistical analysis.

### 2.13. Immunoblot

iSLK.BAC16 cells transfected with siANO1 and the control siASN for 24 h and treated with or without 1 μg/mL doxycycline (Dox) and 1 mM sodium butyrate (NaB) to induce lytic reactivation for 48 h were lysed in Pierce RIPA buffer (Thermo Scientific, Waltham, MA, USA, #89900). Total protein levels for lysates were quantified using the Pierce BCA Protein Assay Kit (Thermo Scientific, #23225) according to the manufacturer’s instructions. For each knockdown condition, 30 μg of total protein lysate was loaded onto a 4–20% 10-well Mini-PROTEAN TGX Gel (Bio-Rad, #4561094) and transferred to PVDF membranes (Bio-Rad, #1620264). Membranes were blocked for 1 h with 5% skim milk in 0.1% TBST (1× Tris-buffered saline containing 0.1% Tween 20) (Thermo Scientific, #28360) and incubated with primary antibodies diluted in 5% skim milk-TBST overnight at 4 °C. Following washing, membranes were incubated with HRP-conjugated secondary antibodies diluted in 5% skim milk-TBST for 2 h at room temperature. Immunoreactive proteins were detected using Clarity Western ECL substrate (Bio-Rad) and an Amersham ImageQuant™ 800 Western blot imaging system (Cytiva, Marlborough, MA, USA). Primary antibodies included mouse anti-ORF45 (Sigma-Aldrich, St. Louis., MO, USA, #SAB5300153, 1:1000) and mouse anti-K8.1 (Millipore, #MABF2678, 1:500). HRP-conjugated goat anti-mouse IgG (Jackson ImmunoResearch, West Grove, PA, USA, #115-035-003, 1:20,000) was used as secondary antibodies. ORF45 and K8.1 band intensities were quantified using Fiji/ImageJ. Protein expression was first normalized to the corresponding total lane protein determined by Ponceau S staining. The Ponceau-normalized values were subsequently normalized to the reactivated siASN (+ASN) control, which was assigned a value of 1 for graphical presentation. Statistical analyses were performed on these normalized biological replicate values using one-way ANOVA. Experiments were performed in three independent biological replicates, and representative immunoblots and their quantification are shown.

### 2.14. Drug Treatment

iSLK.BAC16 cells were seeded in a 6-well plate at a density of 1.65 × 10^5^ cells/well or 50% confluency. The next day, cells were treated with different concentrations of the ANO1-inhibiting drugs, Ani9 (TOCRIS, Avonmouth, Bristol, UK, 6076) and diethylstilbestrol (DES) (Sigma-Aldrich, 46207). At 24 h post-treatment, cells were treated with −/+Dox/NaB to induce lytic reactivation. At 48 h post-lytic reactivation, supernatant was collected and saved to perform titer experiments. The cells were harvested for qPCR analysis or used for flow cytometry analysis.

### 2.15. Extracellular Virus Titers

All iSLK.BAC16 cell supernatants were collected post-experimental treatment and subjected to centrifugation to clear debris at 300× *g* for 10 min. iSLK cells were seeded in 12-well plates at 70% confluency 24 h before infection. 500 μL of supernatant supplemented with polybrene (10 μg/mL) from treated cells was added to iSLK cells.

Infection was enhanced by spinoculation at 300× *g* and 37 °C for 1 h (Acceleration 7, Deceleration 7) and then incubated for 4 h at 37 °C with interval shaking every 30 min. The infectious medium was replaced with fresh iSLK medium after 4 h. Infectivity was assessed 48 h post-infection by imaging GFP^+^ iSLK cells using the BIO-RAD ZOE Fluorescent Cell Imager. GFP+ signal was quantified using Fiji/ImageJ software by measuring the area of GFP+ fluorescence using identical threshold settings across all samples, and statistical analysis was subsequently performed.

### 2.16. Statistical Analysis

Figures and statistical analyses were generated using GraphPad Prism 10.5.0 software. Data are presented as the mean ± SEM from at least three independent biological replicates. For RT-qPCR, Western blot, immunofluorescence, and infectious virus assays, normalized biological replicate values (relative to the +ASN control for siRNA experiments or the no-drug +D/N control for pharmacological inhibition experiments) were used for statistical analyses. Apoptosis data were analyzed using the raw percentages obtained by flow cytometry without additional normalization. Statistical significance between two groups was determined using an unpaired two-tailed Student’s *t*-test. Comparisons among multiple groups were performed using one-way ANOVA followed by the appropriate post hoc multiple-comparison test. Differences were considered statistically significant at *p* < 0.05. Statistical significance is indicated as follows: (*, *p* ≤ 0.05; **, *p* ≤ 0.01; ***, *p* ≤ 0.001; ****, *p* ≤ 0.0001), ns, non-significant.

## 3. Results

### 3.1. vGPCR Overexpression Leads to Global Changes in the Host Cell Transcriptome, Including the Upregulation of ANO1

**Figure 1:**
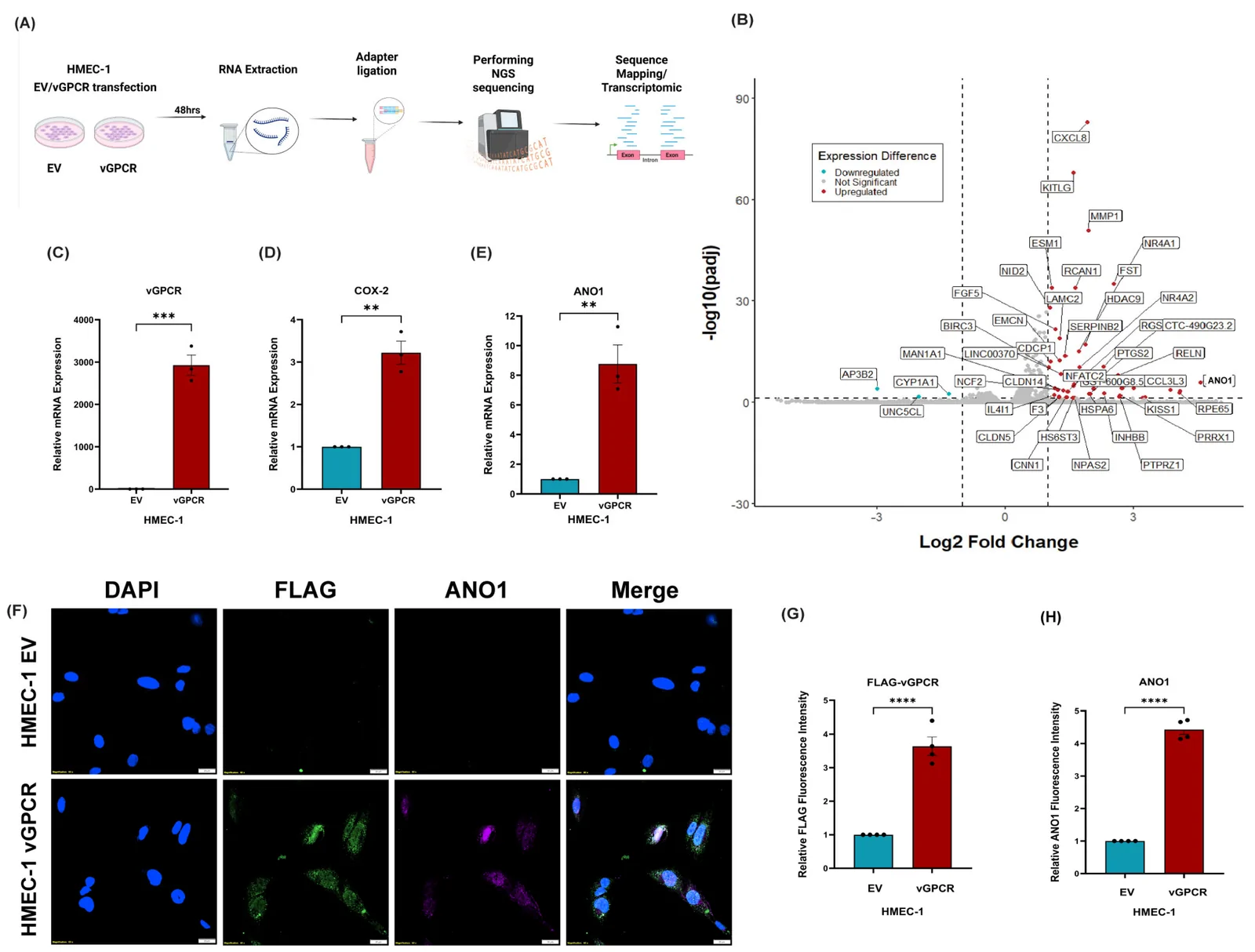
ANO1 is upregulated in vGPCR-expressing endothelial cells. (**A**) Schematic of the bulk RNA-sequencing workflow. HMEC-1 cells were transfected with either an empty vector (EV) or vGPCR plasmid for 48 h prior to RNA extraction for RNA sequencing and RT-qPCR validation. Created in BioRender. Lab, S. (2026) https://BioRender.com/v76cyp3. (**B**) Volcano plot showing differentially expressed host transcripts in vGPCR-expressing HMEC-1 cells compared with EV controls. Differentially expressed genes were defined using an adjusted *p*-value < 0.05 and |log2 fold change| ≥ 1. (**C**–**E**) RT-qPCR validation of (**C**) vGPCR, (**D**) COX-2, and (**E**) ANO1 expression in HMEC-1 cells expressing vGPCR relative to EV controls. Relative transcript levels were normalized to GAPDH and expressed relative to the EV control. (**F**) Representative confocal immunofluorescence images showing FLAG-tagged vGPCR and ANO1 protein expression in EV- and vGPCR-transfected HMEC-1 cells. Nuclei were stained with DAPI. (**G**) Quantification of FLAG fluorescence intensity and (**H**) ANO1 fluorescence intensity normalized to DAPI and expressed relative to the EV control. Data represent mean ± SEM from at least three independent biological replicates for RT-qPCR and four independent biological replicates for immunofluorescence quantification. Statistical significance was determined using an unpaired two-tailed Student’s *t*-test on the normalized biological replicate values. ****, *p* ≤ 0.0001; ***, *p* ≤ 0.001; **, *p* ≤ 0.01. EV, empty vector.

Human microvascular endothelial cells (HMEC-1) were transfected with the SSF-tagged vGPCR plasmid to assess the changes in host gene expression induced by vGPCR compared to the empty vector-transfected cells. A schematic representation of the experimental approach to determine gene expression changes in vGPCR-transfected endothelial cells is shown in Figure 1A. All samples were harvested 48 h after transfection, followed by next-generation sequencing on mRNA libraries from empty vector (EV) and vGPCR samples in triplicate. Out of a total of 37,986 unique transcripts, 37,986 were successfully aligned, and 49 annotated genes were differentially expressed (padj ≤ 0.05, Log2FoldChange > |1|) in empty control vs. vGPCR-transfected cells as depicted in the volcano plot (Figure 1B). Our results show that 3 genes were downregulated, and 46 genes were upregulated, indicating that vGPCR expression and its downstream signaling induce a wide array of transcriptional changes in endothelial cells. Heatmap visualization of all the significantly differentially expressed genes across replicates further illustrated the consistent transcriptional reprogramming in vGPCR-overexpressing HMEC-1 cells (Supplemental Figure S1A).

Among the 46 upregulated genes, several were previously described to be increased in vGPCR-dependent host transcriptional alterations and genes related to the pro-inflammatory and pro-angiogenic pathways, viral lytic induction, and extracellular matrix remodeling. Cyclooxygenase 2 (COX2) [25,26], CXCL8 [17], KITLG [27], and RCAN1 [27] were all upregulated in our RNA-seq analysis (Figure 1B). Downregulated genes included CYP1A1, UNC5CL, and AP3B2. Gene ontology analysis of the top 10 significantly activated pathways revealed enrichment for pathways involved in cell movement and tissue remodeling, and the top 10 significantly suppressed pathways revealed downregulation of pathways associated with translational and ribosome biogenesis machinery, indicating suppression of global translational activity (Supplemental Figure S1B). The expression levels of some of the top upregulated genes in our HMEC-1 RNA-sequencing data, previously studied in vGPCR-dependent transcriptional alterations, including FST, CXCL8, HDAC9, and KITLG, were confirmed using RT-qPCR analysis (Supplemental Figure S1C).

To validate successful vGPCR transfection, we assessed mRNA expression of the canonical vGPCR target gene, COX-2 [25,26], in empty vector and vGPCR-transfected cells. RT-qPCR analysis confirmed that upon overexpression of vGPCR, the relative mRNA expression of COX-2 showed a significant ~3.2-fold increase compared to empty vector controls (Figure 1C,D). Furthermore, several previously unknown putative vGPCR targets were identified to be upregulated significantly (~9-fold increase), notably ANO1, a transmembrane calcium chloride channel implicated in apoptosis inhibition, cell proliferation, and tumor progression [20,21,22] (Figure 1E). Studies show that ANO1 inhibits apoptosis and regulates the cell cycle [28]. We have thereby confirmed the RNA-sequencing data using RT-qPCR and observed that the relative mRNA expression of ANO1 was also significantly upregulated in vGPCR-expressing endothelial cells. Taken together, these results provide strong evidence that the transcriptional changes in HMEC-1 cells are a direct consequence of vGPCR expression and its downstream signaling. Recent studies have shown that ANO1 can modulate host cell apoptosis through similar mechanisms as vGPCR [22,29]. To further validate the expression of the SSF-tagged vGPCR construct and assess its effect on ANO1 protein expression, we performed immunofluorescence staining in EV and vGPCR-transfected HMEC-1 cells. FLAG staining confirmed the expression of SSF-tagged vGPCR in transfected HMEC-1 cells, while ANO1 protein expression was markedly increased in vGPCR-expressing cells compared to EV controls (Figure 1F). The SSF-tagged vGPCR construct displayed predominantly cytoplasmic and membrane-associated localization, consistent with previous reports describing KSHV vGPCR localization to intracellular membrane compartments and the trans-Golgi network (TGN) in transfected and infected cells [30,31]. Quantification of FLAG-tagged vGPCR and ANO1 fluorescence intensity from four independent experiments further confirmed robust expression of SSF-tagged vGPCR and significantly increased ANO1 protein levels in vGPCR-expressing HMEC-1 cells compared to EV controls (Figure 1G,H). Together with the induction of canonical vGPCR-responsive genes, including COX-2, these findings support the conclusion that the SSF-tagged construct retains downstream signaling activity. Collectively, our data reveal that vGPCR drives broad transcriptional remodeling in endothelial cells and identifies ANO1 as a novel vGPCR-responsive gene that may help sustain the apoptosis-resistant phenotype in endothelial cells.

### 3.2. Knockdown of ANO1 Induces Apoptotic Cell Death in HMEC-1 Cells

**Figure 2:**
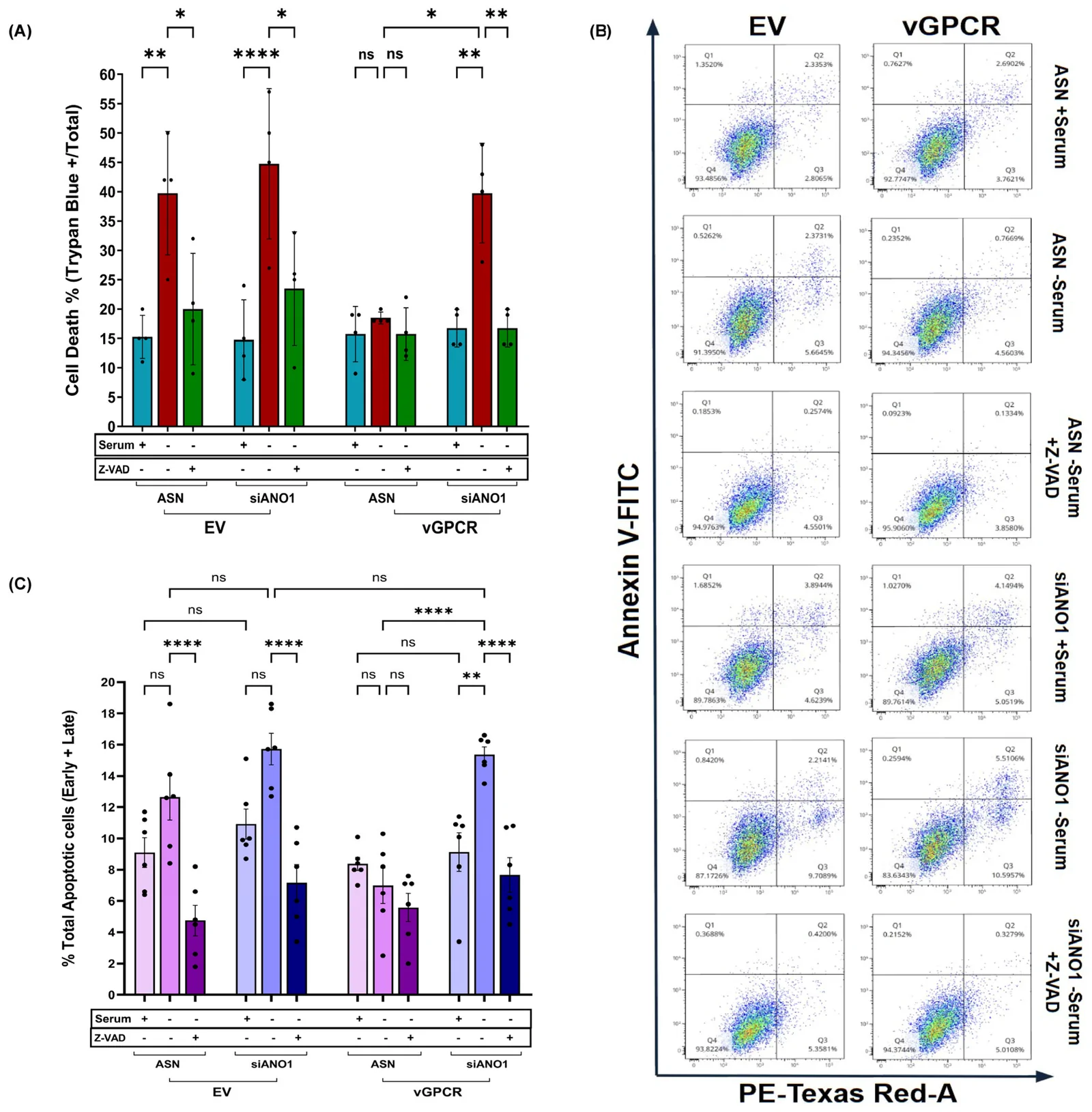
ANO1 knockdown induces caspase-dependent apoptosis in vGPCR-expressing HMEC-1 cells under serum starvation. HMEC-1 cells transfected with vGPCR were treated with siANO1 and cultured under full serum or 12 h serum-starved conditions, with or without the pan-caspase inhibitor Z-VAD-FMK (25 μM). (**A**) Cell death was quantified by trypan blue exclusion assay in HMEC-1 cells transfected with an empty vector (EV) or vGPCR and treated with control siRNA (ASN) or siANO1 under full-serum (+) or serum-starved (–) conditions for 12 h, in the presence or absence of Z-VAD-FMK. Serum starvation significantly increased cell death in EV cells and in vGPCR-expressing cells following ANO1 knockdown, which was rescued by Z-VAD-FMK treatment, indicating caspase-dependent cell death. (**B**) Representative Annexin V-FITC/PI flow cytometry plots of EV and vGPCR-expressing HMEC-1 cells under the indicated conditions. (**C**) Quantification of total apoptotic cells (Annexin V- and/or PI-positive cells) by Annexin V-FITC/PI flow cytometry under the same conditions as in (**A**). vGPCR expression reduced serum starvation-induced apoptosis, whereas ANO1 knockdown significantly increased apoptosis in serum-starved vGPCR-expressing cells. This increase was significantly attenuated by Z-VAD-FMK treatment, supporting a caspase-dependent mechanism. Data are presented as the raw percentages of total apoptotic cells (mean ± SEM), with individual biological replicates shown as dots (*n* ≥ 3 independent experiments). Statistical analyses were performed using raw biological replicate values and analyzed by one-way ANOVA with multiple comparison correction. ****, *p* ≤ 0.0001; **, *p* ≤ 0.01; *, *p* ≤ 0.05; ns, not significant. EV, empty vector; ASN, All-Star Negative siRNA. Generated in GraphPad.

Cell death can occur through several programmed or non-programmed mechanisms, including apoptosis (caspase-driven dismantling of cellular components), necroptosis (RIPK-mediated membrane rupture), autophagic cell death (excessive autophagosome formation), and pyroptosis (inflammasome-activated gasdermin pores) [22,29,32,33,34]. As previous studies have reported that ANO1 can modulate host cell apoptosis through mechanisms similar to those activated by vGPCR [35,36], we sought to test if vGPCR-mediated ANO1 upregulation modulates apoptotic cell death during lytic KSHV infection. To investigate the functional role of ANO1 in vGPCR-mediated cell survival, we measured cell death percentages following ANO1 knockdown under both full serum and serum-starved conditions in HMEC-1 cells. Serum starvation is a well-established method to induce apoptotic cell death by depriving cells of external survival signals, including nutrients and growth factors. This approach is widely used to simulate stress conditions and to assess apoptosis sensitivity in endothelial cells, as previous studies have shown that serum deprivation reliably activates caspase-dependent apoptosis [37,38,39,40]. The knockdown efficiency of ANO1 in vGPCR-transfected HMEC-1 cells was observed to be ~70% (Supplementary Figure S3A). Immunofluorescence analysis further confirmed efficient knockdown of ANO1 protein expression following siANO1 treatment in vGPCR-transfected HMEC-1 cells (Supplementary Figure S3C,D). Under full serum conditions, ANO1 knockdown did not significantly affect cell death in either empty vector or vGPCR-expressing cells, suggesting that ANO1 does not have a role in vGPCR-mediated survival under non-stressed conditions (Figure 2A). In contrast, under serum-starved conditions, a significant difference was observed. vGPCR-expressing cells expressing ANO1 exhibited significantly lower cell death compared to both empty vector controls and vGPCR-expressing cells with ANO1 knockdown. Notably, cell death levels in vGPCR-expressing cells with ANO1 knockdown closely resembled those of serum-starved empty vector controls, indicating that the loss of ANO1 attenuates the protective effect conferred by vGPCR during nutrient deprivation. These results suggest that ANO1 is specifically required for vGPCR-mediated inhibition of apoptosis under serum-starved conditions, indicating ANO1 as a critical mediator of vGPCR-driven cell survival during stress.

To determine whether the observed cell death upon ANO1 knockdown in vGPCR-expressing cells is due to apoptosis, we performed cell viability assays in the presence or absence of the pan-caspase inhibitor Z-VAD-FMK. vGPCR-transfected HMEC-1 cells treated with siANO1 showed a significant increase in cell viability when treated with Z-VAD-FMK compared to DMSO-treated controls (Figure 2A), indicating that the loss of viability was caspase-dependent. This apoptosis rescue effect indicates that the reduced cell viability observed following ANO1 knockdown is caspase-dependent and suggests that ANO1 contributes to vGPCR-mediated suppression of apoptosis in endothelial cells. These results demonstrate that ANO1 is a critical effector of vGPCR-mediated cell survival under stress conditions. Although ANO1 is not required for vGPCR-mediated cell survival under nutrient-rich conditions, it becomes important for maintaining the anti-apoptotic effects of vGPCR during serum starvation. The caspase-dependent nature of cell death following ANO1 knockdown further supports a role for ANO1 in limiting apoptosis under stress conditions. These results support a model in which vGPCR upregulates ANO1 to promote endothelial cell survival during lytic KSHV infection, highlighting ANO1 as a key mediator of vGPCR-driven anti-apoptotic signaling.

To more precisely quantify apoptosis, we repeated the serum starvation experiment by flow cytometry using Annexin V-FITC and PI staining. Consistent with our viability assays, ANO1 knockdown had no effect under full serum conditions, with both EV and vGPCR-expressing cells showing high viability, with ~90% of cells remaining viable (FITC-PI-, Q4) regardless of ANO1 knockdown, while early (Q1) + early-late (Q2) + late apoptotic cells (Q3) (FITC^−^PI^−^ + FITC^+^PI^+^ + FITC^−^PI^+^) remained low, confirming that ANO1 loss does not impact survival under nutrient-rich conditions (Figure 2B,C). However, serum starvation revealed a clear requirement for ANO1 in vGPCR-mediated survival. Serum starvation substantially increased total apoptotic cell populations in EV control cells from approximately 9% under full-serum conditions to ~13% following serum withdrawal. In contrast, vGPCR expression partially protected cells from serum starvation-induced apoptosis, with total apoptotic cells remaining unchanged (~8% in full serum versus ~7% following serum starvation). This protective effect was lost following ANO1 knockdown, resulting in a marked increase in total apoptotic cells, from ~9% total apoptotic cells under full-serum conditions to ~15% following serum starvation. Notably, apoptosis levels in vGPCR + siANO1 cells closely resembled those of serum-starved EV control. Similar to the trypan blue viability assay, treatment with the pan-caspase inhibitor Z-VAD-FMK significantly reduced apoptosis in serum-starved siANO1-treated cells to approximately 7–8%, consistent with the results obtained using the trypan blue viability assay and demonstrating that ANO1 depletion induces caspase-dependent apoptosis. These data confirm that ANO1 is specifically required for vGPCR-dependent inhibition of apoptosis during nutrient stress, consistent with its proposed role as a key mediator of vGPCR-driven anti-apoptotic signaling.

### 3.3. Global Transcriptomic Changes in iSLK.BAC16 Cells During KSHV Lytic Reactivation

**Figure 3:**
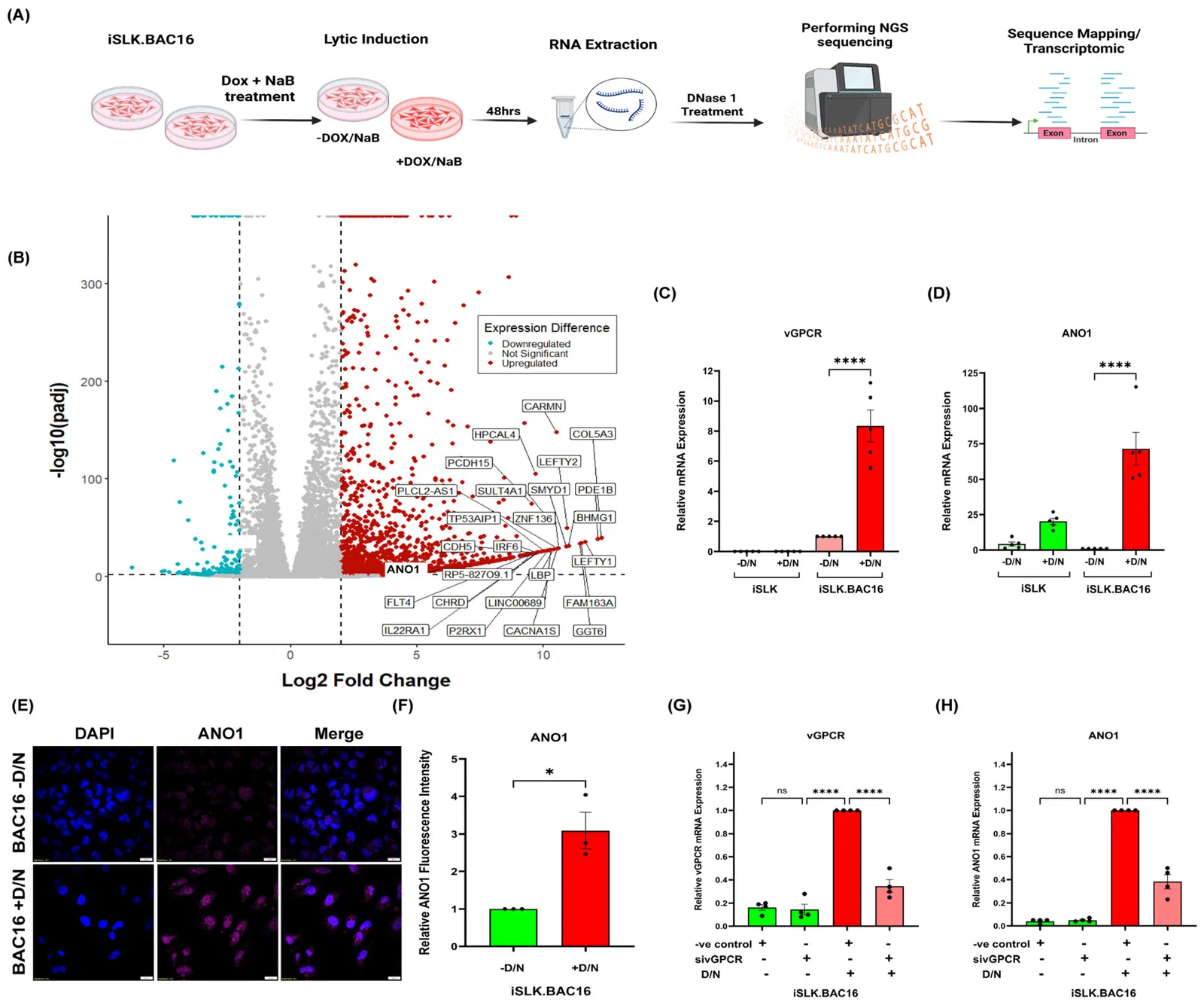
ANO1 is upregulated during KSHV lytic reactivation and is regulated by vGPCR. (**A**) Schematic of the RNA-sequencing workflow used to compare latent and lytically reactivated iSLK.BAC16 cells following doxycycline and sodium butyrate (D/N) treatment for 48 h. Created in BioRender. Lab, S. (2026) https://BioRender.com/vwrgbrr. (**B**) Volcano plot showing differentially expressed host transcripts during KSHV lytic reactivation. Significantly upregulated and downregulated genes are shown in red and blue, respectively. Differentially expressed genes were defined using an adjusted *p*-value < 0.05 and |log2 fold change| ≥ 2. (**C**) RT-qPCR validation of vGPCR expression and (**D**) ANO1 expression in parental iSLK and iSLK.BAC16 cells under latent and lytic conditions. Relative transcript levels were normalized to GAPDH and expressed relative to the non-reactivated iSLK.BAC16 (−D/N) control. (**E**) Representative confocal immunofluorescence images showing increased ANO1 protein expression following lytic reactivation in iSLK.BAC16 cells. Nuclei were stained with DAPI. (**F**) Quantification of ANO1 fluorescence intensity normalized to DAPI and expressed relative to the non-reactivated (−D/N) control. (**G**) RT-qPCR confirming efficient siRNA-mediated knockdown of vGPCR following lytic induction. (**H**) ANO1 mRNA expression following vGPCR knockdown demonstrates that vGPCR contributes to ANO1 upregulation during KSHV lytic reactivation. Relative transcript levels were normalized to tubulin and expressed relative to the negative control siRNA-treated reactivated (+D/N) cells. Data represent mean ± SEM from *n* ≥3 independent biological replicates. Statistical significance was determined by one-way ANOVA using the normalized biological replicate values. ****, *p* ≤ 0.0001; * *p* ≤ 0.05. D/N = Doxycycline/Sodium Butyrate. Genes with adjusted *p*-values of 0.05 and Log2FoldChange of −2 and 2.

To investigate the involvement of host factors in KSHV viral gene expression, we utilized iSLK.BAC16 cells, a well-established cell line that stably carries the BAC16 KSHV genome, which contains a constitutively expressed GFP reporter and expresses the viral lytic switch protein RTA (Replication and Transcriptional Activator) under the control of a doxycycline-inducible promoter [41]. To assess host gene expression during lytic KSHV infection, we measured the expression of ANO1 in iSLK.BAC16 cells before and after the induction of lytic reactivation by RT-qPCR analysis. A schematic representation of the experimental approach to determine gene expression changes in reactivated iSLK.BAC16 compared to the non-reactivated iSLK.BAC16 is shown in Figure 3A. iSLK.BAC16 cells were reactivated using doxycycline (Dox) and sodium butyrate (NaB) for 48 h, and RNA was extracted for next-generation sequencing (RNA sequencing). Gene expression profiles were compared between uninduced (latent) and reactivated (lytic) conditions to identify differentially expressed genes. Out of 47,403 unique transcripts aligned, 3269 genes were found to be significantly differentially expressed using a cutoff (padj < 0.005 and log2FoldChange > |2|). Among these, 3056 genes were upregulated, and 213 were downregulated. Volcano plots visualizing differential gene expression reveal a clear shift toward upregulation of host transcripts during lytic reactivation, suggesting a substantial remodeling of the host transcriptome in response to KSHV lytic gene expression (Figure 3B). Importantly, several of the genes found to be upregulated in vGPCR-overexpressing HMEC-1 cells were also observed to be elevated during KSHV reactivation in iSLK.BAC16 cells. These include HSPA6, PTGS2, RGS7, NCF2, NPAS2, INHBB, HDAC9, PTPRZ1, NR4A1, NID2, IL4I1, ANO1, and HS6ST3. These genes are involved in pathways involving angiogenesis, epithelial cell adhesion, IL18 signaling, and cancer. This convergence reinforces the relevance of the HMEC-1 vGPCR model for studying host responses to KSHV signaling.

To validate the transcriptomic findings, we performed RT-qPCR analysis in latent and reactivated iSLK.BAC16 cells and compared expression levels to those in parental iSLK cells [24]. Following lytic induction with Dox and NaB, vGPCR expression was significantly upregulated in reactivated iSLK.BAC16 cells, showing a ~7-fold increase compared to non-reactivated iSLK.BAC16 and parental iSLK controls (Figure 3C). Similarly, ANO1 expression was significantly increased following lytic reactivation, with an ~70-fold increase relative to non-reactivated iSLK.BAC16 cells (Figure 3D). Notably, treatment of parental iSLK cells with Dox and NaB alone modestly increased ANO1 expression (~20-fold), indicating that reactivation-independent effects of Dox/NaB may partially contribute to ANO1 induction. Consistent with these findings, ANO1 was among the upregulated host genes identified in the lytic iSLK.BAC16 transcriptome (Fold Change = 63.294), suggesting that KSHV may exploit ANO1-mediated pathways during lytic reactivation.

To determine whether increased ANO1 transcript levels were accompanied by increased protein expression, confocal immunofluorescence microscopy was performed. ANO1 protein expression was markedly increased following lytic reactivation, with significantly greater fluorescence intensity observed in reactivated iSLK.BAC16 cells than in latent controls (Figure 3E,F).

To determine whether ANO1 induction during KSHV lytic reactivation is dependent on vGPCR expression, iSLK.BAC16 cells were transfected with siRNA targeting vGPCR prior to lytic induction. Efficient knockdown of vGPCR was confirmed by RT-qPCR (Figure 3G). Importantly, vGPCR knockdown significantly reduced ANO1 expression compared with reactivated cells transfected with the negative control siRNA (Figure 3H), demonstrating that vGPCR contributes to ANO1 upregulation during KSHV lytic reactivation and supporting the conclusion that ANO1 is a downstream host target of vGPCR signaling in the context of infection.

In addition, FABP4 and FABP7, members of the fatty acid-binding protein family involved in lipid metabolism and inflammation, were also upregulated, suggesting a potential link between lipid metabolic reprogramming and viral reactivation [42]. Metabolism-associated pathways were prominently enriched among the upregulated genes, including fatty acid biosynthesis, glycolysis/gluconeogenesis, and oxidative phosphorylation [43,44,45]. This reprogramming is consistent with the metabolic demands of viral replication and may reflect a host attempt to control cellular homeostasis under stress.

Comparison with a previously published RNA-seq dataset of lytically reactivated iSLK.BAC16 cells revealed similar induction of ANO1 and several differentially expressed genes identified in our study, including IRF6, IRF4, PTGS2, ISG15, RUNX3, CXCR4, SOCS3, and JAK2, among several others, supporting the reproducibility of our transcriptomic findings [46]. Several genes previously reported to be modulated during KSHV lytic replication were also differentially expressed in our dataset, including PTGS2 [47,48,49], RUNX3 [50], ISG15 [51], BATF3, IRF4, IRF6 [52], TLR5 [53], CXCR4 [54], SOCS3 [55,56], JAK2 [2,57], and STAT5A [58]. These genes are known to participate in inflammatory signaling, oxidative stress response, and cell survival pathways.

Heatmap visualization of the top 50 upregulated genes across replicates further illustrated the consistent transcriptional reprogramming in reactivated iSLK.BAC16 cells (Supplementary Figure S2A). ANO1 was ranked the 411th upregulated gene among the 3056 total genes upregulated in our differential expression analysis (out of only the significantly upregulated genes; ones with non-significant adjusted *p*-values were not included in the ranking). Gene ontology analysis of the top 10 most significantly suppressed pathways and the top 10 most significantly activated pathways is shown in Supplementary Figure S2B. Differentially expressed genes in latent versus lytic iSLK.BAC16 cells were significantly enriched in GO terms associated with muscle system processes, extracellular matrix components, calcium signaling, and immune regulation, suggesting a coordinated response involving cell morphogenesis, matrix remodeling, and apoptosis-related immune activation. Taken together, our transcriptomic analysis of iSLK.BAC16 cells following KSHV reactivation reveals a robust upregulation of host genes that mirror vGPCR-driven transcriptional programs, including inflammatory cytokines [17,59,60], pro-survival factors [26,61,62], and metabolic regulators [63,64]. These results provide further evidence that vGPCR contributes to shaping the host response during lytic replication and that targets such as ANO1 may serve as novel mediators of KSHV pathogenesis.

### 3.4. Knockdown and Pharmacological Inhibition of ANO1 Induce Apoptosis During KSHV Infection

Because our earlier findings demonstrated that ANO1 promotes survival of vGPCR-expressing endothelial cells under stress, we next asked whether ANO1 regulates apoptosis during KSHV infection. ANO1 has also been implicated in regulating cell survival pathways in cancer and viral infection contexts [22,29]. To test whether ANO1 influences apoptotic responses during KSHV reactivation, we performed siRNA-mediated knockdown of ANO1 in iSLK.BAC16 cells, followed by lytic induction with Dox and NaB. The knockdown efficiency of ANO1 in reactivated iSLK.BAC16 cells was observed to be ~75% (Supplementary Figure S3B). Under latent conditions, basal ANO1 expression was low, and ANO1 knockdown efficiency was limited.

**Figure 4:**
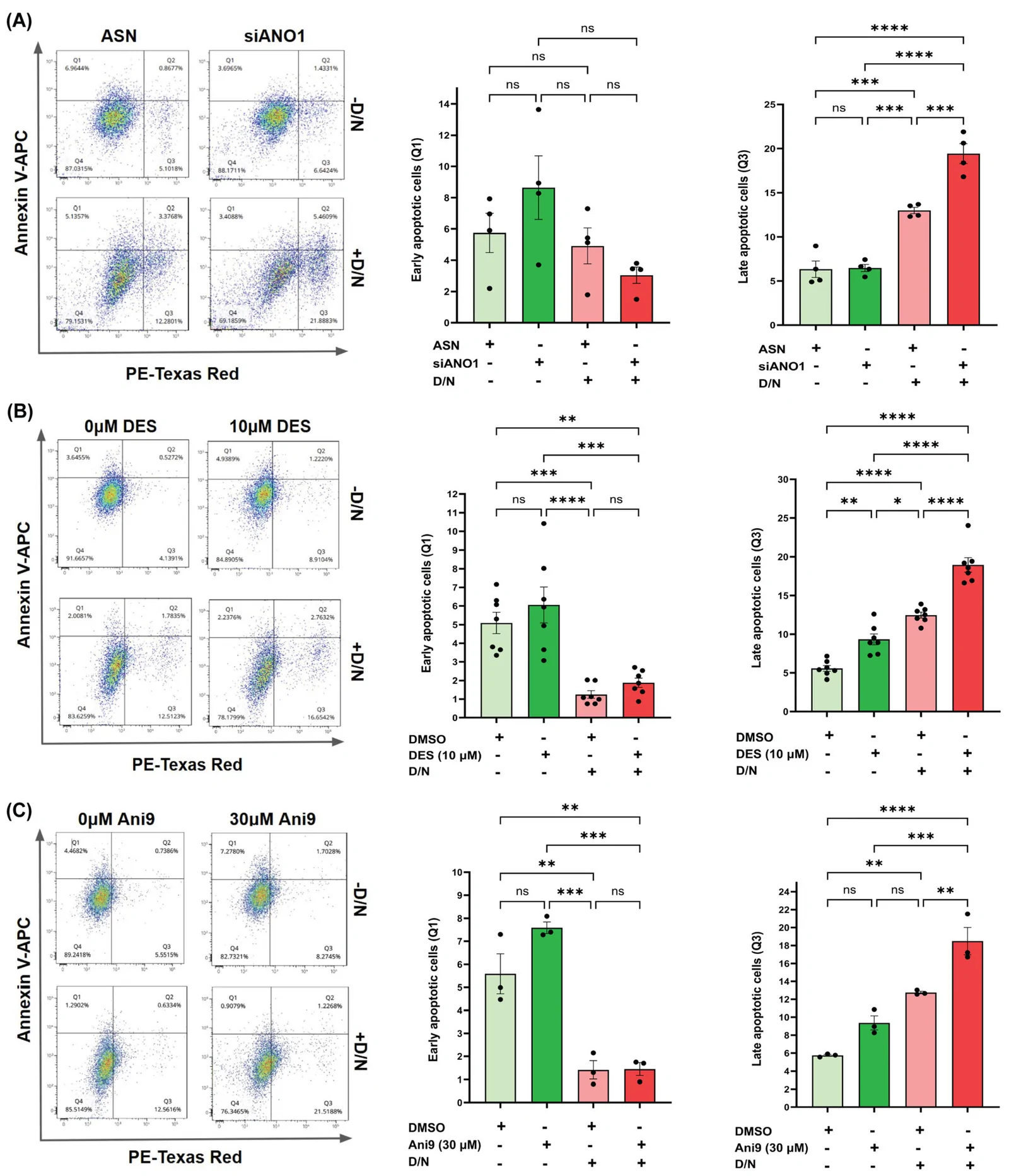
siRNA knockdown or pharmacological inhibition of ANO1 increases late apoptosis during KSHV lytic reactivation. iSLK.BAC16 cells were transfected with siANO1 or treated with the ANO1 inhibitors diethylstilbestrol (DES, 10 μM) or Ani9 (30 μM), followed by lytic induction with doxycycline and sodium butyrate (D/N) for 48 h. Apoptosis was measured by flow cytometry using Annexin V-APC and Propidium Iodide (PI) staining to distinguish early and late apoptotic populations. A significant increase in late apoptotic cells in reactivated lytic samples but not in latent cells was measured upon (**A**) siRNA-mediated knockdown of ANO1, (**B**) DES treatment, and (**C**) Ani9 treatment. While a significant increase in late apoptotic cells was measured, early apoptotic cells remained unchanged in all conditions. Data are presented as the raw percentages of apoptotic cells. Bars represent mean ± SEM from [*n* ≥ 3] independent experiments; individual biological replicates are shown as dots. Statistical analyses were performed using raw biological replicate values and analyzed by one-way ANOVA with multiple comparison correction (****, *p* ≤ 0.0001; ***, *p* ≤ 0.001; **, *p* ≤ 0.01; *, *p* ≤ 0.05; ns, not significant). D/N = Doxycycline/Sodium Butyrate; ASN = All-Star Negative.

We measured apoptosis by flow cytometry using Annexin V-APC and propidium iodide (PI) staining to distinguish early and late apoptotic populations. In cells transfected with siANO1, no significant differences were observed in the percentage of early apoptotic cells compared with ASN controls under either latent or lytic conditions (Figure 4A). In contrast, late apoptotic cells increased from approximately 13% to 19% following ANO1 knockdown during lytic reactivation, whereas no significant change was observed in latent cells. These findings suggest that ANO1 suppresses apoptosis during KSHV lytic replication. The greater apoptotic response observed during lytic reactivation is consistent with the substantially higher ANO1 expression following viral reactivation and the more pronounced effect of ANO1 depletion under these conditions. Lytic induction with doxycycline and sodium butyrate alone also produced a modest increase in late apoptotic cells compared with latent controls.

To further determine whether ANO1 depletion influences caspase activation during lytic KSHV infection, activated caspase-3 was measured by flow cytometry in iSLK.BAC16 cells following siRNA-mediated ANO1 knockdown in the presence or absence of Dox/NaB (Supplementary Figure S5). Under non-reactivated conditions, ANO1 knockdown significantly increased activated caspase-3 compared with the ASN controls. KSHV lytic reactivation also significantly increased activated caspase-3 compared with non-reactivated control cells. As a control for apoptosis induction, we treated cells with staurosporine, an apoptosis-inducing agent. As expected, treatment with staurosporine markedly increased caspase-3 activation [65] Overall, these findings indicate that ANO1 depletion in iSLK.BAC16 cells promotes caspase activation.

Pharmacological inhibition of ANO1 using selective inhibitors DES (diethylstilbestrol) or Ani9 has been reported in connection with apoptosis regulation [66,67]. DES is a selective ANO1 inhibitor that induces apoptosis in various cancer cells by increasing caspase-3 activity and PARP-1 cleavage, with effective concentrations generally around 10 μM, showing anticancer effects without hepatotoxicity at this dose. DES has been previously shown to reduce both ANO1 channel activity and cell viability, reduce p-ERK1/2 and p-EGFR levels, and induce apoptosis by increasing caspase-3 activity and PARP-1 cleavage; however, it does not affect the ATP-induced increase in intracellular Ca^2+^ levels [66]. Ani9 is a potent and highly selective small-molecule ANO1 inhibitor with an IC50 below 3 μM, previously used at concentrations ranging from 10 to 30 μM to robustly block ANO1 channel activity and induce apoptosis in prostate cancer cells without affecting related chloride channels or intracellular calcium signaling [66,67,68,69,70]. These inhibitors have been consistently shown to increase apoptosis in ANO1-expressing cells, supporting their authenticity and functional relevance in studying the role of ANO1 in cell survival and death. DES and Ani9 treatment aligns with previous research demonstrating efficacy in inducing apoptosis through ANO1 inhibition, which complements the genetic knockdown approach by confirming the role of ANO1 in modulating apoptotic pathways.

iSLK.BAC16 cells were treated with the selective ANO1 inhibitors DES (10 μM) and Ani9 (30 μM) in the presence or absence of Dox and NaB induction of reactivation. Both drugs significantly increased apoptosis compared to vehicle controls, with reactivated cells showing a higher apoptotic rate than untreated or latent cells. Upon DES treatment, a significant increase in late apoptotic cells was observed under both latent (~6% to 9%) and lytic (~13% to 19%) conditions; however, the effect was markedly more pronounced during lytic reactivation (Figure 4B). This suggests that ANO1 inhibition by DES compromises cell survival even in the absence of reactivation stimuli, but the apoptotic response is further amplified once lytic gene expression is induced. Similarly, Ani9 treatment also led to elevated late apoptotic populations upon reactivation (~13% to 18%), while the early apoptotic cells remained unchanged during lytic reactivation, confirming that disruption of ANO1 function enhances apoptotic cell death during KSHV reactivation (Figure 4C). This suggests that ANO1 inhibition induces a significant increase in late apoptosis of cells undergoing lytic reactivation. Our flow cytometry analysis also revealed that Dox and NaB treatment alone modestly increased the proportion of late apoptotic cells during the knockdown or pharmacological inhibition of ANO1, consistent with apoptosis associated with viral reactivation.

### 3.5. ANO1 Inhibition Promotes Early and Late KSHV Lytic Gene Expression

**Figure 5:**
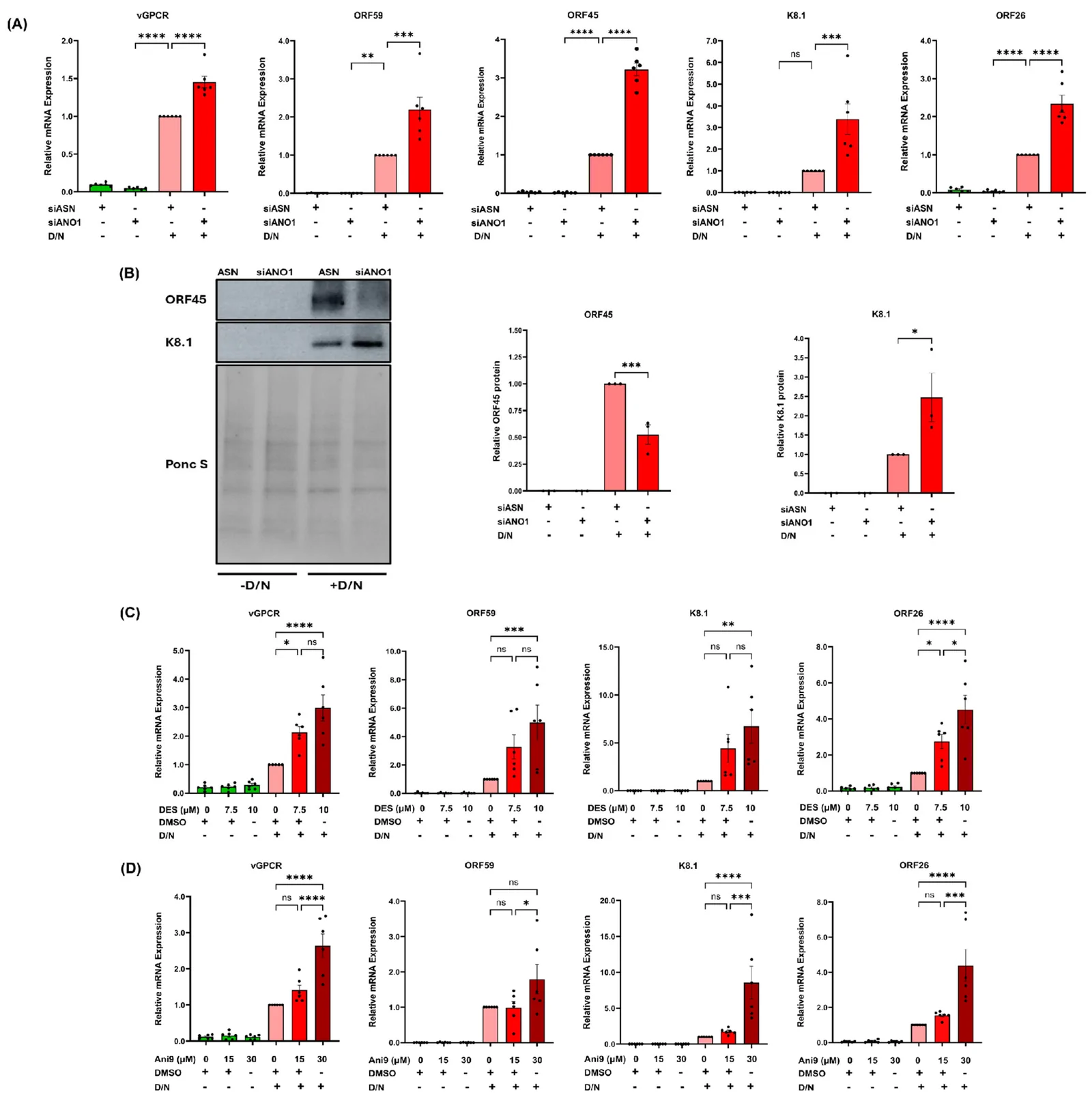
ANO1 knockdown or pharmacological inhibition enhances KSHV lytic gene expression during viral reactivation. iSLK.BAC16 cells were transfected with siANO1 or treated with ANO1 inhibitors diethylstilbestrol (DES) (7.5 μM or 10 μM) or Ani9 (15 μM or 30 μM), followed by lytic induction with doxycycline and sodium butyrate (D/N) for 48 h. (**A**) RT-qPCR analysis of the immediate-early/early lytic genes vGPCR, ORF59, and ORF45, and late lytic genes K8.1 and ORF26, following siRNA-mediated ANO1 knockdown. (**B**) Representative immunoblots of ORF45 and K8.1 protein expression following ANO1 knockdown, with Ponceau staining shown as the total protein loading control. Quantification of ORF45 and K8.1 protein levels normalized to total lane protein is shown to the right. (**C**) RT-qPCR analysis of lytic gene expression following DES treatment. (**D**) RT-qPCR analysis of lytic gene expression following Ani9 treatment. All RT-qPCR data were normalized to the housekeeping gene Tubulin and subsequently expressed relative to the reactivated +ASN control (**A**) or the no-drug +D/N control (**C**,**D**), which was assigned a value of 1. ORF45 and K8.1 protein expression was quantified by normalizing band intensities to the corresponding total lane protein determined by Ponceau staining and subsequently expressed relative to the reactivated +ASN control. Data represent mean ± SEM from six independent biological replicates for RT-qPCR and three independent biological replicates for immunoblot quantification. Statistical significance was determined by one-way ANOVA using the normalized biological replicate values. ****, *p* ≤ 0.0001; ***, *p* ≤ 0.001; **, *p* ≤ 0.01; * *p* ≤ 0.05; ns, non-significant. D/N = Doxycycline/Sodium Butyrate; ASN = All-Star Negative.

To determine whether ANO1 regulates KSHV lytic reactivation, ANO1 expression was inhibited in iSLK.BAC16 cells by siRNA-mediated knockdown or pharmacological inhibition with Ani9 or DES. Cells were reactivated with doxycycline and sodium butyrate (Dox/NaB) for 48 h, and expression of representative KSHV lytic genes was assessed by RT-qPCR. Following ANO1 knockdown, expression of the immediate-early/early lytic genes vGPCR, ORF59, and ORF45 was significantly increased by ~0.5-fold, ~1.5-fold, and ~2.5-fold, respectively, compared with the siASN control after lytic induction (Figure 5A). Likewise, the late lytic genes K8.1 and ORF26 were significantly upregulated by ~2.5-fold and ~1.5-fold, respectively, indicating enhanced progression through the lytic replication program. These findings suggest that ANO1 depletion promotes KSHV lytic gene expression. To determine whether these transcriptional changes were reflected at the protein level, ORF45 and K8.1 protein expression was examined by immunoblotting. Consistent with the RT-qPCR results, ANO1 knockdown increased K8.1 protein expression. In contrast, although ORF45 transcript levels were increased, intracellular ORF45 protein levels were reduced following ANO1 knockdown (Figure 5B).

Treatment with Ani9 and DES resulted in a dose-dependent increase in both early and late lytic gene expression. Treatment with lower concentrations of DES (7.5 μM) resulted in a non-significant increase in lytic gene expression; notably, higher doses (10 μM DES and 30 μM Ani9) resulted in a significant increase in both early lytic, vGPCR (~2-fold increase) and ORF59 (~4-fold increase), and late lytic, K8.1 (~6.5-fold increase) and ORF26 (~4-fold increase) gene expression compared to the no-drug control (Figure 5B). Similarly, treatment with lower concentrations of Ani9 (15 μM) resulted in a non-significant increase in lytic gene expression, but higher doses (30 μM Ani9) resulted in a significant increase in both early lytic, vGPCR (~1.5-fold increase) and ORF59 (~0.5-fold increase), and late lytic, K8.1 (~7-fold increase) and ORF26 (~3.5-fold increase) gene expression compared to the no-drug control (Figure 5C). These results suggest a dose-dependent effect of ANO1 inhibition on KSHV reactivation. Strikingly, all three ANO1-targeting approaches, siANO1, DES, and Ani9, produced the same outcome: a marked increase in the early and late lytic gene expression. Together, these findings indicate that ANO1 acts as a modulator of lytic gene expression, and its inhibition potentially promotes viral reactivation.

### 3.6. ANO1 Inhibition Enhances the Production of Infectious KSHV Particles

**Figure 6:**
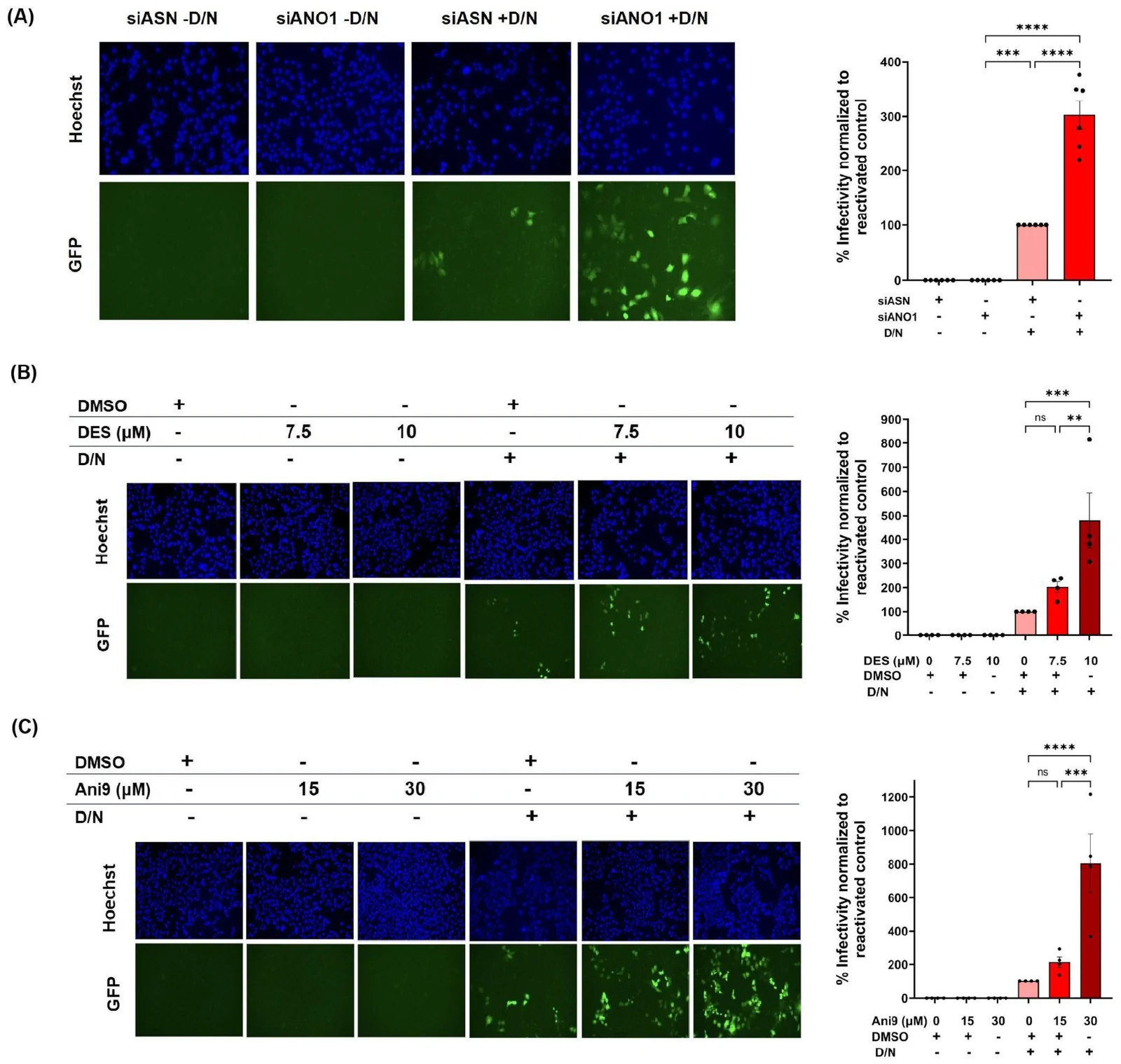
siRNA knockdown or pharmacological inhibition of ANO1 leads to increased extracellular viral titers. Cell-free supernatants from iSLK.BAC16 cells after knockdown or inhibition of ANO1 were collected after reactivation, and viral titers were determined. GFP+ cells correspond to KSHV-infected cells. Titers were assessed at 48 hpi on a ZOE fluorescent microscope at 10×. (**A**) Lytic reactivation and siRNA-mediated knockdown of ANO1 significantly increased the production of infectious extracellular KSHV particles when titered on iSLK cells. Quantification of GFP+ cells observed in (**A**). (**B**,**C**) Pharmacological inhibition of ANO1 using Ani9 (**B**) and DES (**C**) resulted in a dose-dependent increase in extracellular viral titers, with higher concentrations promoting a significant increase in viral release. Imaged using ZOE, Fiji/ImageJ was used to quantify the relative % of infectivity to reactivated control (siASN + D/N or DMSO + D/N). Generated in GraphPad. Data represent mean ± SEM from [*n* ≥ 3] independent experiments. Statistical significance was determined by one-way ANOVA using biological replicate values normalized to the appropriate reactivated control (+ASN for siRNA experiments or the no-drug +D/N control for pharmacological inhibition experiments); ****, *p* ≤ 0.0001; ***, *p* ≤ 0.001; **, *p* ≤ 0.01; ns, non-significant. D/N = Doxycycline/Sodium Butyrate; ASN = All-Star Negative.

To assess whether the host gene, ANO1, influences the release of infectious KSHV particles, we measured viral titers following ANO1 knockdown and pharmacological inhibition during lytic reactivation. We performed siRNA-mediated knockdown/inhibition of ANO1 in iSLK.BAC16 cells followed by lytic reactivation. We collected the cell-free KSHV-containing supernatants from siANO1 and drug-treated samples and titered them on iSLK cells. We determined the infection levels in iSLK cells by quantification of GFP+ cells via fluorescence microscopy. Our results show a significant increase in the production of infectious virions following ANO1 knockdown, as determined by the elevated percentage of GFP+ iSLK cells after infection with supernatants collected from reactivated siANO1-treated iSLK.BAC16 cells, with a ~300% increase in infection compared to ASN controls (Figure 6A). Similarly, treatment with DES or Ani9 significantly increased infectious virion production, resulting in a ~500% and ~800% increase in GFP+ iSLK cells, respectively, compared to vehicle-treated controls (Figure 6B,C). Notably, DES and Ani9 treatments did not result in an increase in viral titers at lower concentrations (7.5 μM DES and 15 μM Ani9) but significantly increased the infectious particles with higher concentrations of the drugs (10 μM DES and 30 μM Ani9).

As convergent inhibition of ANO1 by three independent methods consistently enhanced early and late gene expression (Figure 5) and increased infectious virion release (Figure 6), these findings suggest that loss of ANO1 promotes infectious virion production, enhancing KSHV lytic replication and the release of infectious virus. Given our earlier observations that ANO1 loss promotes late apoptosis during KSHV infection (Figure 4), the increase in extracellular virus titers upon ANO1 inhibition may be mechanistically linked to apoptotic membrane remodeling or vesicle release pathways that facilitate viral release, suggesting that cellular stress and ANO1 dysfunction create a permissive environment for productive viral release.

## 4. Discussion

Here, we demonstrate that vGPCR expression in endothelial cells induces transcriptional reprogramming, including the robust upregulation of the calcium-activated chloride channel ANO1. Both RNA-sequencing and RT-qPCR analyses confirm that ANO1 is among the most significantly elevated host genes in vGPCR-expressing HMEC-1 cells (Figure 1B,E), alongside previously known vGPCR targets such as COX-2 and CXCL8. Our findings align with reports that viral manipulation of endothelial cells in diseases such as Kaposi’s sarcoma involves the induction of pro-angiogenic and inflammatory genes and the suppression of vascular homeostasis signatures [71,72,73,74]. Importantly, ANO1 upregulation was also observed in reactivated iSLK.BAC16 cells, a model of KSHV reactivation (Figure 3B,D). Consistent with these transcriptomic findings, immunofluorescence analysis confirmed that ANO1 protein expression was also significantly increased following lytic reactivation (Figure 3E,F), demonstrating that transcriptional induction is accompanied by increased protein abundance. ANO1 upregulation during KSHV lytic reactivation was also reported in a previously published RNA-seq dataset of reactivated iSLK.BAC16 cells, supporting the reproducibility of ANO1 induction during KSHV lytic reactivation [46]. However, despite these transcriptomic observations, the functional role of ANO1 during KSHV infection has not previously been investigated. Our study extends these findings by demonstrating that vGPCR contributes to ANO1 upregulation during KSHV lytic reactivation, as siRNA-mediated depletion of vGPCR significantly reduced ANO1 expression (Figure 3G,H), and that ANO1 functionally contributes to apoptosis resistance, viral gene expression, and infectious virion production.

Functional studies reveal that ANO1 is a critical effector of vGPCR-mediated cell survival under nutrient-limiting conditions. While ANO1 depletion did not affect viability in nutrient-rich conditions, serum starvation revealed a strict dependence on ANO1 for vGPCR to suppress apoptosis (Figure 2B). Rescue of ANO1 knockdown-induced cell death by the pan-caspase inhibitor Z-VAD-FMK demonstrates that the loss of viability is caspase-dependent, consistent with the reported anti-apoptotic role of ANO1 in cancers [22,29]. These findings suggest that ANO1 functions as an important mediator of the pro-survival phenotype associated with vGPCR expression during cellular stress [35,36].

Interestingly, knockdown or pharmacological inhibition of ANO1 in KSHV-infected iSLK.BAC16 cells not only enhanced apoptotic cell death (Figure 4) but also increased lytic gene expression (Figure 5) and the production of extracellular infectious virions (Figure 6). Together, these results suggest that ANO1 is required to suppress late-stage apoptosis during KSHV lytic replication, supporting a role for this calcium-activated chloride channel in sustaining host cell survival to optimize viral replication, while its loss appears to favor viral gene expression, late-lytic protein expression, and virion production, implicating ANO1 as a key modulator of host–virus interactions.

To further examine the involvement of apoptotic caspases upon the loss of ANO1, activated caspase-3 was assessed following ANO1 depletion by siRNA (Supplementary Figure S5). ANO1 knockdown in iSLK.BAC16 cells increased caspase-3 activation in reactivated samples compared to non-reactivated controls. Unexpectedly, we did not observe a further increase in caspase-3 activation in reactivated siANO1 cells compared to ASN control, suggesting a more complex viral-host interaction may be occurring in reactivated iSLK.BAC16 cells compared to our vGPCR-overexpression model. This observation is interesting given the complex interplay between KSHV lytic proteins and the cellular caspase machinery. KSHV K5 has been reported to protect lytically infected cells from caspase-mediated cell death, while ORF57 is subjected to caspase-7-mediated cleavage, which alters ORF57 function and restricts viral lytic gene expression [75,76]. More recently, ORF57 has also been shown to regulate host and viral transcription through the protooncogene FOS and RGS2-associated AKT phosphorylation [46]. Together, these observations suggest that caspase activity during KSHV reactivation is integrated with multiple viral regulatory mechanisms. It is therefore possible that viral modulation of caspase signaling during lytic replication contributes to the different effects of ANO1 depletion observed under latent and reactivated conditions. However, whether K5, ORF57, or other KSHV proteins directly contribute to the ANO1-associated apoptotic phenotype remains to be determined.

Interestingly, ANO1 knockdown increased ORF45 transcript abundance but reduced intracellular ORF45 protein levels, whereas K8.1 protein expression increased (Figure 5B). Because ORF45 is a tegument protein incorporated into mature virions, one possibility is that ANO1 depletion enhances progression through late lytic replication and virion production, thereby altering the intracellular pool of ORF45 through increased packaging into newly formed particles and/or enhanced protein turnover. However, this interpretation remains speculative and would require analysis of ORF45 in purified extracellular virions and protein-stability assays.

The observed increase in viral titers following ANO1 loss could be related to apoptosis-associated membrane remodeling or vesicle release mechanisms that facilitate virion release [77,78]. Such coupling between apoptosis regulation and viral replication strategy has been documented in other herpesviruses and may represent a critical node in KSHV pathogenesis. In KSHV, one mechanism involves the latency-associated nuclear antigen (LANA) restraining p53-dependent apoptosis, thereby preserving the survival of infected cells [79]. This strategy parallels our observation that vGPCR-induced ANO1 upregulation in endothelial and KSHV-infected cells inhibits apoptosis, suggesting that ANO1 may be an additional host target co-opted to prolong cell viability during infection.

However, our caspase-3 activation data indicate that the relationship between ANO1, apoptosis, and KSHV replication during lytic reactivation is unlikely to be explained solely by increased caspase-3 activation. Consistent with this complexity, apoptotic caspases have been reported to promote viral replication or egress in several viral systems, suggesting that apoptotic signaling can, under certain contexts, facilitate viral dissemination [80]. In our study, ANO1 depletion generated a more pro-apoptotic cellular environment that coincided with increased lytic gene expression, increased late-lytic protein expression, and infectious virus production, although the precise mechanism underlying this relationship remains unclear. Additionally, KSHV v-cyclin binds CDK6 to promote cell cycle progression but also causes phosphorylation that inactivates cellular Bcl-2, promoting apoptosis. The viral Bcl-2 homolog protects against this effect, highlighting how KSHV balances apoptosis to benefit its life cycle [81]. Together, these findings position ANO1-mediated apoptosis inhibition within a broader network of KSHV strategies to fine-tune host survival, balancing latency and lytic reactivation for optimal replication dynamics. Because KSHV lytic induction efficiency varies between independent reactivation experiments, some variability in the magnitude of apoptosis and lytic gene expression was expected; however, the direction of the ANO1-dependent phenotype was consistent across independent biological replicates.

Previous studies report that ANO1 is highly expressed in various types of cancers [21,36,69,82,83]. Knockdown of ANO1 expression in cancer cells leads to a significant amount of cell death, indicating that ANO1 expression is crucial in promoting cancer cell viability [22,83]. A recent study shows that ANO1 expression can modulate the activation of the Akt pathway, a classical pathway that controls a plethora of host physiological processes like apoptosis, metabolism, and cell survival. Not only can Akt activation lead to the inhibition of apoptosis through the activation of the nuclear factor kappa light chain enhancer of activated B cells (NF-kB), but it can also induce cell survival pathways by activating serum/glucocorticoid-regulated kinase (SGK) [22,84,85,86]. Furthermore, they show that the loss of ANO1 expression led to Akt inactivation and increased cell death, indicating that the activation of Akt is key to ANO1-mediated inhibition of host cell death [22]. Previous studies show that KSHV and vGPCR downstream signaling can also lead to the activation of the Akt pathway and NF-kB, which inhibits host cell apoptosis [35,87]. Another group demonstrated that KSHV-driven tumorigenesis is associated with pronounced epigenetic reprogramming, including hypomethylation and upregulation of oncogenic pathways such as PI3K-Akt signaling, underscoring Akt pathway activation as a key feature of KSHV-induced sarcomagenesis [88]. Given these prior observations, our hypothesis is that vGPCR upregulates ANO1, and ANO1 contributes to the maintenance of PI3K-Akt signaling, thereby suppressing caspase-dependent apoptosis under stress. Loss of ANO1 would therefore reduce Akt signaling (or its downstream anti-apoptotic outputs), lowering the apoptotic threshold and allowing caspase activation during serum starvation or during KSHV lytic reactivation.

Although studies have shown that vGPCR-mediated inhibition of cell death is dependent on the activation of the Akt pathway, the interplay between ANO1 and the Akt pathway during the KSHV lytic phase is currently unknown [35]. Future experiments should determine the role of ANO1 in the activation of the Akt pathway during the KSHV lytic phase. The observation that ANO1 is induced in both vGPCR-expressing HMEC-1 cells and reactivated iSLK.BAC16 cells suggest that ANO1 may contribute to additional aspects of KSHV biology beyond anti-apoptotic signaling. ANO1 is a calcium-activated chloride channel whose function directly intersects with calcium homeostasis [89]. Given that vGPCR signaling activates PLCβ, leading to IP3-mediated calcium release [18,35], and that calcium signaling influences both apoptotic cascades [90] and viral reactivation in herpesviruses [91], ANO1 may serve as a connecting pathway linking vGPCR-driven calcium fluxes to downstream survival and reactivation pathways. The enrichment of calcium signaling-related GO terms in our iSLK.BAC16 dataset further supports this connection (Supplementary Figure S2B).

While our findings identify ANO1 as a regulator of apoptosis and KSHV lytic replication, the precise molecular mechanism linking ANO1 to these processes remains to be determined. Although our data demonstrate that vGPCR contributes to ANO1 upregulation during lytic reactivation, additional studies will be required to define the downstream signaling pathways through which ANO1 influences viral gene expression and infectious virion production. Furthermore, validation of these findings in primary endothelial cells and in vivo models will be important to establish their relevance to KSHV pathogenesis. Future studies should investigate the mechanistic interplay between ANO1 activity, calcium signaling, and caspase regulation in KSHV-infected endothelial cells. Pharmacological modulation of calcium flux and genetic dissection of downstream calcium-dependent kinases or phosphatases could clarify whether the ANO1 anti-apoptotic effect depends on calcium-dependent survival signaling or simply on chloride conductance. Similarly, examining specific caspase family members involved in ANO1-dependent survival will be critical to determining whether this pathway converges on intrinsic mitochondrial apoptosis or extrinsic death receptor pathways.

**Figure 7:**
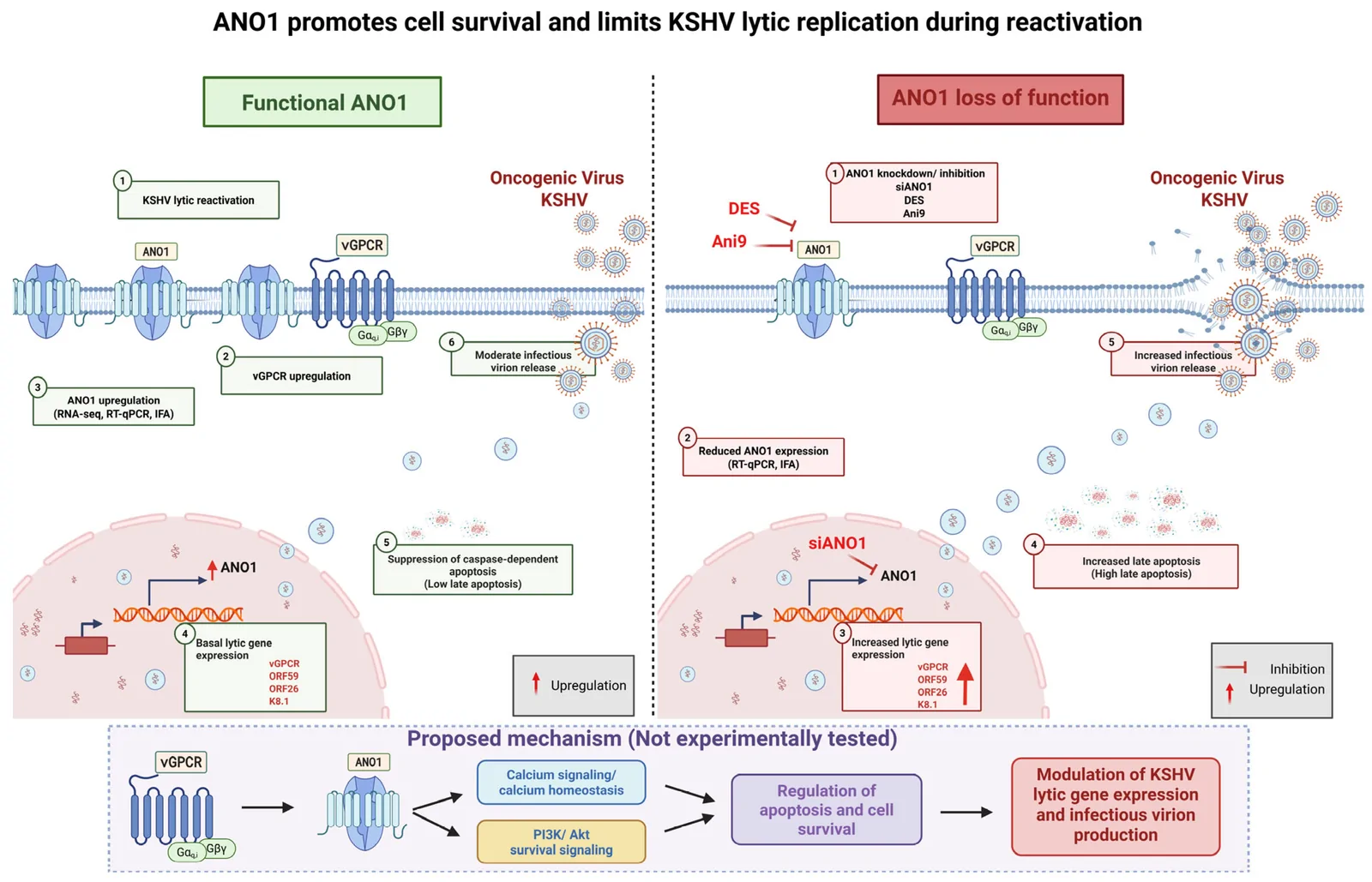
ANO1 modulates apoptosis and virion release during KSHV lytic infection. During KSHV infection, expression of the vGPCR is induced in the lytic phase, leading to transcriptional upregulation of the host calcium-activated chloride channel ANO1. Elevated ANO1 expression helps maintain cell survival by suppressing apoptosis, thereby supporting sustained viral replication within infected cells. In contrast, inhibition or depletion of ANO1 either by siRNA-mediated knockdown or pharmacological inhibitors DES and Ani9 disrupts this protective effect, resulting in enhanced apoptotic cell death. Interestingly, this increased apoptosis coincides with a significant rise in the release of infectious KSHV virions, suggesting that ANO1 acts as a host restriction factor that limits virion egress during lytic replication. Collectively, these findings reveal that ANO1 functions as a key regulator of the cellular environment during KSHV lytic infection, balancing apoptosis and viral release to influence the efficiency of KSHV replication. Created in BioRender. Lab, S. (2026) https://BioRender.com/g3d7b0t.

In conclusion, our findings identify ANO1 as a key vGPCR-induced host factor that supports endothelial cell survival under stress and modulates KSHV lytic replication and infectious virion production (Figure 7). By integrating transcriptional profiling with functional assays, we identify a previously unrecognized role for ANO1 in regulating apoptosis and lytic reactivation during KSHV infection and highlight calcium signaling and caspase-dependent apoptosis as promising avenues for future therapeutic development. Given the ability of ANO1 inhibitors to both induce apoptosis and enhance viral reactivation, careful consideration of the timing and context of targeting ANO1 will be essential for translating these findings into anti-viral or anti-tumor strategies.

## Figures and Tables

**Table 1:** RT-qPCR Primer Sequences.

Target	Model	Forward Primer	Reverse Primer
vGPCR	KSHV	GCTCCTGGGTATCTGCCTAAA	CAGCACCACAGCAACAATCA
ORF59	KSHV	GACAGCGTCTCGCTGACAGA	CACACGCGTGAGCTATTCGG
ORF45	KSHV	CAA CTC TCC GGA CGT GAA CA	GGA GAT TGG GTT GGG AGG TG
K8.1	KSHV	TAACCATGAGTTCCACACAGATTC	GGTTTTGTGTTACACTATGTAGG
ORF26	KSHV	AGCCGAAAGGATTCCACCATT	CCGTGTTGTCTACGTCCAGA
COX-2	Human	GTTCCACCCGCAGTACAGAA	AGGGCTTCAGCATAAAGCGT
ANO1	Human	GATCCCATCCAGCCCAAAGTG	CGGGTTTTGCTGTCGAAAAAGGA
FST	Human	AGCAGCCAGAACTGGAAGTCCA	CCGATTACAGGTCACACAGTAGG
CXCL8	Human	TCTGCAGCTCTGTGTGAAG	TGTGTTGGCGCAGTGTGG
HDAC9	Human	GGAGCAGAAACTGGAGCAG	CAATGATGTGTGGTGGGC
KITLG	Human	CTGGAGACTCCAGCCTACACTG	CTGCCCTTGTAAGACTTGGCTG
GAPDH	Human	CTGCCCAAGATCTTCCATGT	GACAAGCTTCCCGTTCTCAG
Tubulin	Human	TCCAGATTGGCAATGCCTG	GGCCATCGGGCTGGAT

## Data Availability

Data is contained within the article or Supplementary Materials.

## Supplementary Materials

The following supporting information can be downloaded at: https://www.mdpi.com/article/10.3390/v18090954/s1, Supplementary Figure S1. vGPCR overexpression leads to global changes in the host cell transcriptome. (A) Heatmap showing all of the differentially expressed cellular transcripts, including 6 lncRNAs in vGPCR-transfected cells compared to EV controls. (B) Gene Ontology presenting the top 10 most significantly activated pathways and the top 10 most significantly suppressed pathways. (C) RT-qPCR validation of some of the top upregulated genes, previously studied in vGPCR-dependent transcriptional alterations. Generated in GraphPad. Data represent mean ± SEM from [*n* = 3] independent experiments. Statistical significance was determined by *t*-test; ***, *p* ≤ 0.001; *, *p* ≤ 0.1. Genes with adjusted *p*-values < 0.05 and Log2FoldChange > |1| were plotted. EV, Empty Vector. Supplementary Figure S2. Global transcriptomic changes in iSLK.BAC16 cells during KSHV reactivation. (A) Heatmap visualization of the top 50 upregulated genes across replicates in iSLK.BAC16 cells (B) Gene Ontology presenting the top 10 most significantly activated pathways and top 10 most significantly suppressed pathways in iSLK.BAC16 non-reactivated versus Dox/NaB reactivated iSLK.BAC16 cells. Supplementary Figure S3. Knockdown efficiency of ANO1 in HMEC-1 and BAC16 cells. (A) RT-qPCR analysis showing approximately 70% knockdown of ANO1 mRNA expression in vGPCR-transfected HMEC-1 cells following siANO1 treatment compared to All-Star Negative (ASN) control cells. RNA-sequencing analysis revealed minimal ANO1 expression in EV-transfected HMEC-1 cells prior to vGPCR overexpression (normalized FPKM: EV = 2.04; vGPCR = 41.47). (B) RT-qPCR analysis showing approximately 70% knockdown of ANO1 mRNA expression in iSLK.BAC16 cells following siANO1 treatment during lytic reactivation. (C) Representative immunofluorescence images demonstrating ANO1 protein expression in EV- and vGPCR-transfected HMEC-1 cells following ASN or siANO1 treatment. Nuclei were stained with DAPI. (D) Quantification of ANO1 fluorescence intensity from four independent biological replicates confirms significant reduction in ANO1 protein expression following siANO1 treatment in vGPCR-transfected HMEC-1 cells. Fluorescence intensity was quantified in Fiji/ImageJ using identical threshold settings across all samples. Data represent mean ± SEM from ≥ 3 independent experiments. Statistical significance was determined by Student’s *t*-test or one-way ANOVA as indicated; ****, *p* ≤ 0.0001; ns, not significant. ASN, All-Star Negative; D/N, doxycycline/sodium butyrate. Supplementary Figure S4. Kill curve analysis of ANO1 inhibitors in iSLK.BAC16 cells. (A) iSLK.BAC16 cells were treated with increasing concentrations of DES (0–15 μM) or DMSO control, and cell death was quantified by the trypan blue exclusion assay. DES concentrations used in subsequent experiments (≤10 μM) did not induce significant cell death, whereas higher concentrations resulted in increased toxicity. (B) iSLK.BAC16 cells were treated with increasing concentrations of the selective ANO1 inhibitor Ani9 (0–45 μM) or DMSO control, and cell death was assessed by the trypan blue exclusion assay. Ani9 concentrations used in downstream experiments (≤30 μM) did not significantly affect cell viability, while higher doses induced increased cell death. Data are presented as mean ± SEM from independent experiments (*n* ≥ 3). Statistical significance was determined using one-way ANOVA: *** *p* < 0.001, ** *p* < 0.01, * *p* < 0.05, and ns, not significant. Supplementary Figure S5. ANO1 depletion increases caspase-3 activity in iSLK.BAC16 cells. iSLK.BAC16 cells were transfected with AllStars negative control siRNA (ASN) or siRNA targeting ANO1 (siANO1) and maintained under non-reactivated (−Dox/NaB) or lytically induced (+Dox/NaB) conditions. Staurosporine (S) was included as a positive control for caspase activation. Caspase-3 activity was assessed using the cell-permeable fluorogenic caspase-3 inhibitor Red-DEVD-FMK followed by flow cytometry. (A) Representative flow cytometry plots showing Red-DEVD-FMK fluorescence under the indicated conditions. (B) Quantification of the percentage of cells exhibiting activated caspase-3. ANO1 knockdown significantly increased caspase-3 activation under non-reactivated conditions compared with ASN controls. KSHV lytic induction also significantly increased caspase-3 activation in ASN-treated cells; however, ANO1 knockdown did not further significantly increase caspase-3 activation during lytic induction. Staurosporine treatment resulted in robust caspase-3 activation and served as a positive control. Data are presented as mean ± SEM from three independent experiments. Statistical significance is indicated as *** *p* < 0.001 and **** *p* < 0.0001; ns, not significant.

## Abbreviations

The following abbreviations are used in this manuscript:

KSHV	Kaposi’s Sarcoma Herpes Virus
KS	Kaposi’s Sarcoma
ANO1	Anoctamin-1
ORF59	Viral DNA Polymerase
K8.1	Viral glycoprotein
vGPCR	Viral G-Protein Couples Receptor
DES	Diethylstilbestrol
HMEC-1	Human microvascular endothelial cells
hpi	Hours post induction
